# Yeast mismatch repair components are required for stable inheritance of gene silencing

**DOI:** 10.1371/journal.pgen.1008798

**Published:** 2020-05-29

**Authors:** Qian Liu, Xuefeng Zhu, Michelle Lindström, Yonghong Shi, Ju Zheng, Xinxin Hao, Claes M. Gustafsson, Beidong Liu

**Affiliations:** 1 Department of Chemistry and Molecular Biology, University of Gothenburg, Medicinaregatan, Goteborg, Sweden; 2 Institute of Biomedicine, University of Gothenburg, Goteborg, Sweden; 3 Department of Biology, Functional Biology, KU Leuven, Heverlee, Belgium; 4 Center for Large-scale cell-based screening, Faculty of Science, University of Gothenburg, Medicinaregatan, Goteborg, Sweden; University Medical Centre Groningen and University of Groningen, NETHERLANDS

## Abstract

Alterations in epigenetic silencing have been associated with ageing and tumour formation. Although substantial efforts have been made towards understanding the mechanisms of gene silencing, novel regulators in this process remain to be identified. To systematically search for components governing epigenetic silencing, we developed a genome-wide silencing screen for yeast (*Saccharomyces cerevisiae*) silent mating type locus *HMR*. Unexpectedly, the screen identified the mismatch repair (MMR) components Pms1, Mlh1, and Msh2 as being required for silencing at this locus. We further found that the identified genes were also required for proper silencing in telomeres. More intriguingly, the MMR mutants caused a redistribution of Sir2 deacetylase, from silent mating type loci and telomeres to rDNA regions. As a consequence, acetylation levels at histone positions H3K14, H3K56, and H4K16 were increased at silent mating type loci and telomeres but were decreased in rDNA regions. Moreover, knockdown of MMR components in human HEK293T cells increased subtelomeric *DUX4* gene expression. Our work reveals that MMR components are required for stable inheritance of gene silencing patterns and establishes a link between the MMR machinery and the control of epigenetic silencing.

## Author summary

During aging, gene silencing also decreases and it has been hypothesized that the collapse of epigenetic control networks may in part explain age-related diseases. For example, changes in epigenetic silencing are linked with different stages of tumor formation and progression. Great efforts have been made on investigating the mechanisms of establishment and maintenance silencing at silent mating cassettes in yeast. In this work, by applying a genome-wide silencing screening approach, we identified the conserved subunits of the mismatch repair (MMR) machinery (Pms1, Mlh1 and Msh2) as new components of the epigenetic silencing regulation machinery in yeast. We also found that depletion of mismatch repair subunits (Mlh1 and Msh2) led to impaired telomere-length related expression in mammalian cells. This indicates that these components probably have an evolutionarily conserved role on influencing gene silencing from yeast to humans. Further studies the functional roles of these MMR components on epigenetic silencing in mammalian model systems or relevant cancer patient samples will increase our understanding of MMR-related oncogenesis.

## Introduction

Chromatin structure alterations help to establish gene silencing, which in part explains heritable gene expression patterns. Changes in epigenetic silencing are associated with different stages of tumour formation and progression [1, 2]. Gene silencing decreases during ageing, and researchers have hypothesized that cancer may, in part, result from an age-related collapse of epigenetic control networks [1, 3]. The mechanisms on establishment and maintenance of gene silencing have been studied in detail in budding yeast silent mating cassettes, *HML* (homothallic mating left) and *HMR* (homothallic mating right) (for reviews, see [4]). Establishment of silencing at these sites is dependent on the DNA sequences E-silencer and I-silencer, which flank *HML* and *HMR* and contain binding sites for Rap1, Abf1, and the origin recognition complex (ORC). The silencer-binding proteins in turn recruit Sir (Silent Information Regulator) proteins that form heterochromatin and prevent transcription of the silent mating cassettes (for reviews, see [5]). Sir3 and Sir4 were found to interact with Rap1 at these loci[6]. Sir2 (a histone deacetylase) and Sir4 can form a stable complex, which recruits Sir3 when positioned at the silencer. The assembled Sir complex spreads via a network of multivalent interactions between Sir3 and Sir4 and de-acetylated lysines in the N-terminal tails of histones H3 and H4 [7]. Mechanistically similar (but less robust) silencing occurs at the telomeres, Sir3 and Sir4 were also found to associate with RAP1 at the telomeres, and Rap1 and yKu70 proteins recruit the Sir2, Sir3 andSir4 complex to establish the chromatin-mediated gene repression at yeast telomeric regions [8, 9]. Thus, silencing at these loci requires the recruitment of Sir2 to the correct genomic locations [10–12]. The Sir proteins are essential for establishing and maintenance silencing at *HML* and *HMR*, and mutations in *SIR2*, *SIR3*, or *SIR4* cause a complete loss of mating ability due to a loss of HM repression [13, 14]. Other genes required to establish silencing at mating cassettes, including *ABF1*, *ORC2*, and *ORC5*, have been identified using a sensitized genetic screen [15]. The complete loss of silencing phenotype of such strains cannot be detected with a synthetic genetic array (SGA) based approach [16, 17], due to the requirement of proper mating ability for constructing the final output strains carrying both the silencing marker and the corresponding gene deletion. In yeast (*S*. *cerevisiae*), *MATa* or alpha mating type information are normally present at *HMR* or *HML respectively*. These two loci are differently regulated. The yeast *SIR1* gene was identified from the observation that *sir1-1* partially loss the silencing at the silent mating type loci[13]. Sir1 was found to be required for the establishment of silencing at *HML* and *sir1Δ* cells can form two mitotically stable states at *HML*. Importantly, the mating and non-mating states of *SIR1*-deficient cells are mitotically stable and thus heritable through successive cell divisions in genetically identical cells [18]. The mating-type cassette silencing phenotype of *SIR1* mutants is therefore a classical example of epigenetically inherited gene silencing, and this kind of silencing phenotype can be used as a readout for an SGA-based screening (which acquires strain’s proper mating ability) for identifying new genes affecting this process.

The mismatch repair (MMR) pathway corrects base-base mispairs and insertion/deletion mispairs that accumulate during normal DNA replication. Key components of the MMR in yeast are the Pms1, Mlh1, and Msh2 proteins [19]. In eukaryotic cells, mispairs are primarily recognized by the Msh2-Msh6 heterodimer, which further recruits the Pms1-Mlh1 complexes to mispairs. The Pms1-Mlh1 complex functions as a DNA endonuclease that nicks the double-stranded DNA, which is followed by excision of the strand with the incorrect base. Mutation of MMR genes cause increased DNA mutation rates and are observed in many types of cancers [20–22]. Whether the MMR pathway has a functional role in maintaining proper gene silencing is currently unknown.

In the current work, we established a genome-wide screening approach to identify genes that exhibit partial loss of silencing due to changes in the epigenetically controlled repression state of the silent *HMR* locus. Our screen identified that the genes *PMS1*, *MLH1*, and *MSH2*, which encode crucial components of MMR, as required for transcriptional repression at silent mating-type loci and telomeres. We observed that MMR deletions affect the occupancy of the silent complex recruiting components on the silent mating-type loci and telomeres, and altered localization of Sir2. Eventually, these changes influence the epigenetic silencing at these loci.

## Results

### Genome-wide genetic screening identifies MMR complex components as required for epigenetic silencing at mating-type cassettes

Yeast synthetic genetic array (SGA) is widely used for genome-wide identification of components involved in biological pathways [23]. The use of SGA to search for novel epigenetic silencing regulators is hindered because the selection markers tagged to the query genes must be properly expressed to enable a series of selection steps in the procedure. In screening for components that are required for epigenetic silencing, the selection marker must be inserted into the silent loci on the genome. This makes SGA inapplicable for this type of study. To achieve our goal of identifying epigenetic silencing regulation components in a genome-wide fashion, we had to overcome two challenges. First, we had to construct a query strain that carries markers in the silent mating-type loci. Second, we had to make the silencing markers selectable in the SGA selection steps, including the selection of diploids and of the final regulatory components.

Usually, the gene expression in the silent mating-type loci is almost completely repressed under the control of Sir2, which makes it almost impossible to insert any markers or select positive transformants on selection plates. To efficiently introduce the *URA3 marker* into the silent *HMR* locus and the *HphR* (hygromycin B phosphotransferase gene, its expression confers hygromycin resistance to *HphR* transformed cells) marker into the *HML* locus, we began by inserting the markers in *SIR2*-deletion background, in which genes at the silent loci are expressed due to loss of silencing as described in our previous work [24]. This makes it possible to insert markers into these loci using the regular PCR knockout approach and enables the selection of positive clones on the corresponding selection plates. After acquiring the positive transformants with the *URA3* and *HphR* markers in these loci, we transferred the wild-type (WT) *SIR2* sequence (without any selection markers) back into its original locus. Positive transformants were selected from plates containing 5-fluoro-orotic acid (5-FOA), a fluorinated derivative of the pyrimidine precursor orotic acid. Yeast cells with an active *URA3* gene convert 5-FOA to fluorodeoxyuridine, which is toxic to cells. This enables the selection of strains that do not express *URA3* by using plates with 5-FOA. We designed this indirect selection method because positive transformants in which a wild-type SIR2 sequence is successfully inserted and properly expressed can fully restore silencing at the *HMR* locus. This means that the expression of the *URA3* marker inserted in the HMR locus will be fully repressed, rendering the strains resistant to 5-FOA.

The query strain was crossed with an ordered array of about 4261 viable yeast deletion mutants (SGA-v2)[16, 17]. In diploid and final triple selection steps, it was necessary to select diploid or *MATa* single-deletion strains that carry the silent *URA3* and *HphR* marker in the *HMR* and *HML* loci. Because genes within these loci are completely silent, it was necessary to temporarily release the repression on these loci by adding nicotinamide (5 mM) to the medium. Nicotinamide is an inhibitor of Sir2 [25]. As a by-product of the Sir2 histone deacetylation reaction, it can generate a negative feedback inhibition on Sir2 activity when reaches a high local concentration in the cell [26].

**Fig 1:**
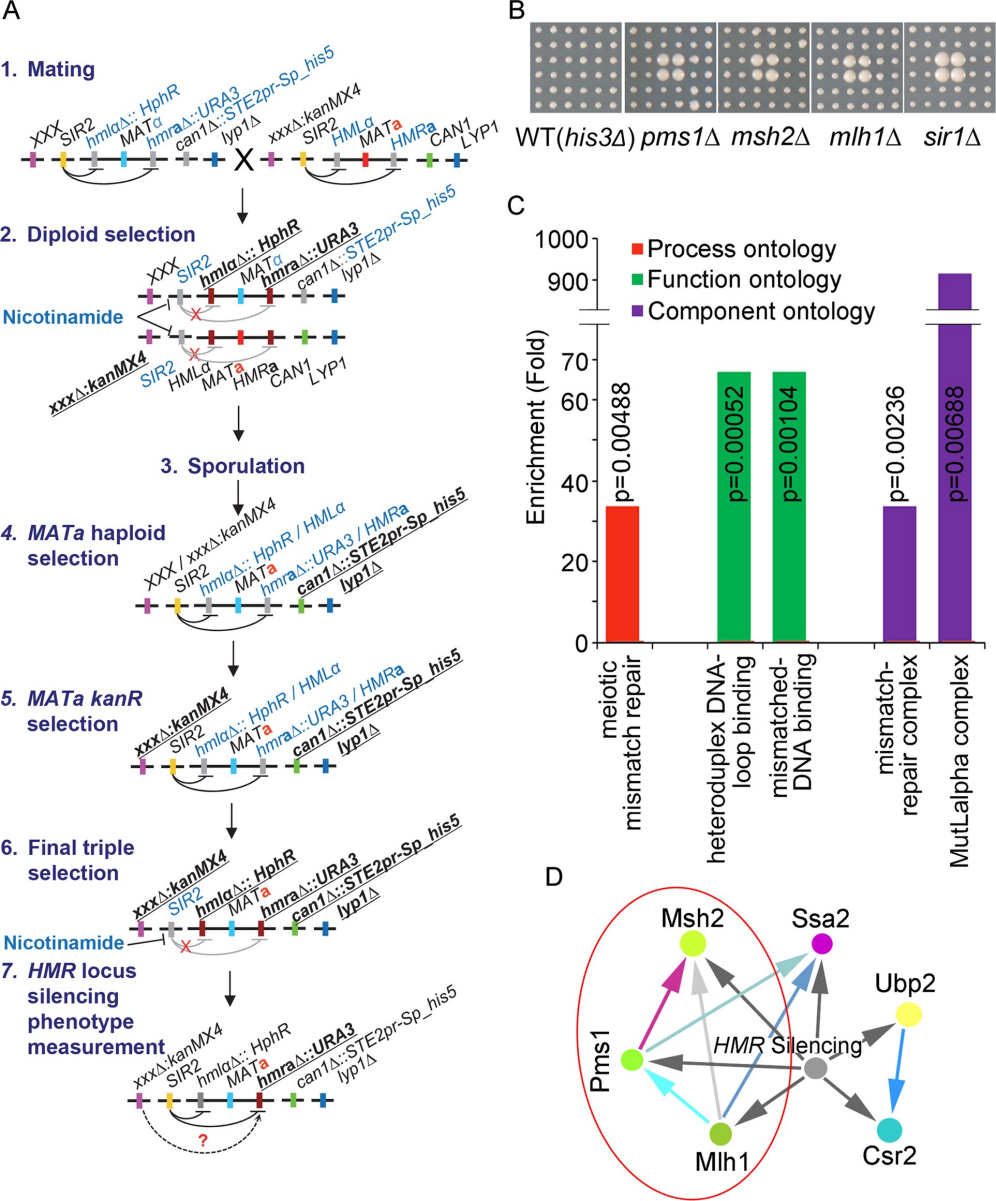
Genome-wide silencing screen identifies MMR complex components as novel candidates of mating-type epigenetic silencing regulation. **(A)** Schematic of the genome-wide silencing screen procedure. Relevant genes or markers are indicated as colour bars with their gene names. Silent or repressed gene names are shown as grey bars and indicated in light blue font. Marker genes, which were selected in each selection step, are indicated using underlined font. **(B)** Deletion of MMR component genes *PMS1*, *MLH1*, and *MSH2* resulted in decreased silencing phenotypes on the agar-based Ura dropout screening plates; *sir1Δ*, which served as a positive control, was also isolated from the screen. **(C)** The MMR pathway-related genes were enriched according to a Gene Ontology (GO) analysis. Biological process, molecular function, and cellular component ontology groups were analysed. **(D)** Physical interaction analysis of the identified hits revealed that Pms1, Mlh1, and Msh2 physically interact with one another. Physical interactions between hits were extracted from the BioGRID interaction database.

Using this approach, we facilitated the selection of the diploid and the target triple mutants carrying the silencing markers (*URA3* and *HphR*) and the yeast single-gene deletion marker (*KanMX4*). After the final triple mutants were acquired, the cells were brought back to their normal silent state from the previous triple selection plates by not including nicotinamide in the medium of the next step. In the last step of the screen, the effect of yeast gene deletion on epigenetic silencing at the *HMR* locus was tested on a medium lacking uracil (SD-Ura) (to simplify the screening procedure, the silencing at the *HML* locus was not measured). We aimed to isolate yeast gene-deletion strains that could grow on the Ura dropout plates; because the wild-type strain cannot grow at all on this selection medium, any strains with colonies larger than those of the WT were selected as potential loss-of-silencing hits. The increased-growth phenotype indicated a decreased silencing at the locus (with a higher expression level of the *URA3* marker than in the WT) due to deletion of the corresponding genes. The SGA based crossing procedure is illustrated in Fig 1A and a complete flow diagram of the overall screening procedure is shown in S1A Fig. Mutants with decreased mating silence were scored based on differences in colony size between deletion mutants and the WT on the selection plates. The screen was performed in duplicate in a 1536-spot format with four replicates for each deletion strain. A total of 413 candidate genes showed a decreased silencing phenotype in the genome-wide silencing screen (S3 Table).

We confirmed that 179 mutants that showed a decreased-silencing phenotype in SD-Ura liquid medium using the Bioscreen mini-liquid culture approach (S4 Table). We further verified 45 hits with decreased-silencing phenotypes that could be clearly observed using conventional spot tests on SD-Ura agar plates (S1B Fig and S5 Table). The *SIR1* mutant was identified as one of the strongest hits. The identification of the *SIR1* mutant served as a positive control and showed that our screening approach could isolate components involved in the regulation of epigenetic silencing. A Gene Ontology (GO) analysis of the 45 hits showed that the meiotic mismatch repair bioprocess category, the mismatched-DNA binding and heteroduplex DNA-loop binding functional categories, and mismatch-repair complex and MutLalpha complex cellular component were significantly enriched (Fig 1C). We found that three of four MMR complex components (i.e., Pms1, Mlh1, and Msh2, but not Msh6) were identified as the strongest hits (Fig 1B and S1B and S2A Figs). We further analysed the physical interaction among these 45 hits, the physical interactions between hits were extracted from the BioGRID interaction database [27], and genes whose products physically interacted are indicated in Fig 1D. The MMR components were found to be tightly linked and to physically interact with one another. Overall, the genome-wide screening results suggested that the MMR complex may have a previously unknown function in the regulation of epigenetic silencing at mating-type cassettes.

### MMR components affect silencing at both mating-type cassettes and telomere loci, and the loss of mating-type silencing was not dependent on the increased mutation rate in the MMR mutants

**Fig 2:**
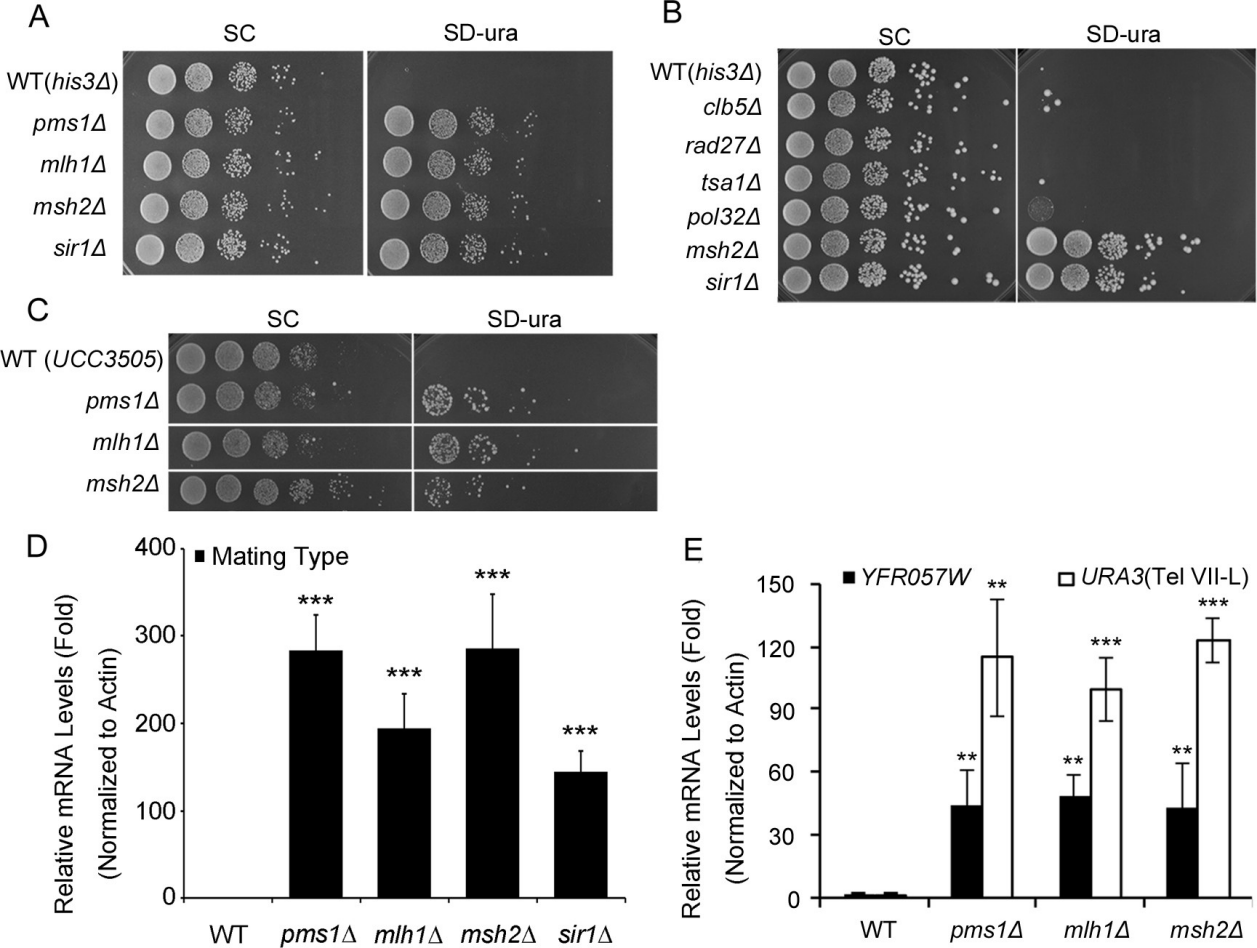
MMR components affect the silencing at both mating-type and telomere loci. **(A)** MMR deletion strains (WT(*his3Δ*), *pms1Δ*, *mlh1Δ*, and *msh2Δ*) showed decreased mating-type cassette silencing. Ten-fold dilutions of the deletion mutants were plated on minimal selective medium lacking uracil (SD-Ura, right panel). Cells grown on minimal complete medium (SC) served as a control (left panel). *sir1Δ* was used as a positive loss-of-silencing control. **(B)** Unlike MMR mutants, mutants with increased DNA mutation rates (*clb5Δ*, *rad27Δ*, *tsa1Δ*, and *pol32Δ*) do not exhibit a loss-of-silencing phenotype; *msh2Δ* and *sir1Δ* served as positive controls. **(C)** Loss of *PMS1*, *MLH1*, and *MSH2* leads to decreased silencing at telomeres. WT(UCC3505) and the corresponding MMR deletion mutants in the UCC3505 background were constructed and tested. Five-fold dilutions of the strains (WT(UCC3505), *pms1Δ*, *mlh1Δ*, and *msh2Δ*) were plated on SD-Ura agar medium (right) and SC medium (left). **(D)** Relative *URA3*-*HMR* mRNA expression levels in WT(*his3Δ*), *pms1Δ*, *mlh1Δ*, and *msh2Δ*. **(E)** Relative *URA3(Tel VII-L) and YFR057W* mRNA expression levels in WT(UCC3505) and *pms1Δ*, *mlh1Δ*, and *msh2Δ* in the UCC3505 background. Fold changes were calculated by normalization to the expression of actin. Experiments were performed in triplicate, and statistical significance was determined using two-tailed Student’s *t*-tests; *, **, and *** indicate significance at *P* < 0.05, < 0.01, and < 0.001, respectively.

We manually validated the mating-type silencing effect of MMR complex mutants (Fig 2A) and reconstructed the deletion strains using a different selection marker (natMX4) using the PCR knockout approach (S2B Fig). We have also tested the deletion mutant of MSH6 using the same marker, and *msh6Δ* did not show the same decrease of silencing phenotype as in the other MMR mutants (S2C Fig). This could be due to that the function of Msh6 is partially redundant with Msh3 in the *MSH2*-dependent mismatch repair process[28]. Deletion of *SIR1* disrupted epigenetic silencing at mating type loci and presented metastable silencing phenotype. We also tested MMR mutants on–URA and +FOA plates and found that MMR mutants also lead to metastable silencing phenotype (S2D Fig). Because deletion of MMR proteins increases DNA mutation rates and consequently leads to generate suppressors [29, 30], the growth phenotype observed on the silencing marker selection plates may have been simply due to suppressors produced by increased mutation rates in the MMR mutant strains. To test this possibility, we selected four other deletion mutants (*clb5Δ*, *rad27Δ*, *tsa1Δ*, and *pol32Δ*) that are also known to have increased DNA mutation rates [31]. None of the four mutants exhibited a loss-of-silencing phenotype similar to that of the MMR mutants (Fig 2B), indicating that the loss of mating-type silencing phenotype observed in the MMR mutant strains was not linked to an increase in the number of suppressors.

Because the maintenance of silencing involves some similar components at silent mating-type loci and telomeres [11, 32, 33], we investigated whether loss of *PMS1*, *MLH1*, and *MSH2* also affected silencing at telomeres. Indeed, we observed a moderate increase in the expression of the *URA3* marker, which was inserted next to the (TG1–3)n repeat region of telomere 7L (Fig 2C). We also measured *URA3* transcription levels at mating-type and telomere loci[34, 35]. The transcript levels were significantly increased at both the mating-type and telomere loci (Fig 2D and 2E). To confirm whether MMR mutants affect endogenous telomere silencing, we measured the transcription level of sub-telomeric *YFR057W* gene[36, 37] and observed significant increase in the mutants (Fig 2E). Beside *URA3* marker, there existed an *ADE2* telomeric reporter in the UCC3505 strain. We therefore also monitored red/red or white sectored colonies formation and the MMR deletion strains (*pms1Δ*, *mlh1Δ*, and *msh2Δ*) displayed white with sectors colonies formation as compared to WT, indicating a loss of gene silencing at the telomere ADE2 reporter. (S2E Fig). Finally, we overexpressed exogenous *MSH2* to complement the telomere and mating type loss of silencing phenotype in the *MSH2* deletion strains. As we expected, overexpression of exogenous *MSH2* partially restore the silencing at both telomere and mating type loci (S2F Fig). In summary, our results suggested that MMR components affected silencing at both mating-type and telomere loci and that the loss of mating type silencing was not dependent on the increased mutation rate in the MMR mutant strains.

### MMR deletions affect the occupancy of the silent complex recruiting components on the HMR and HML loci and telomeres

**Fig 3:**
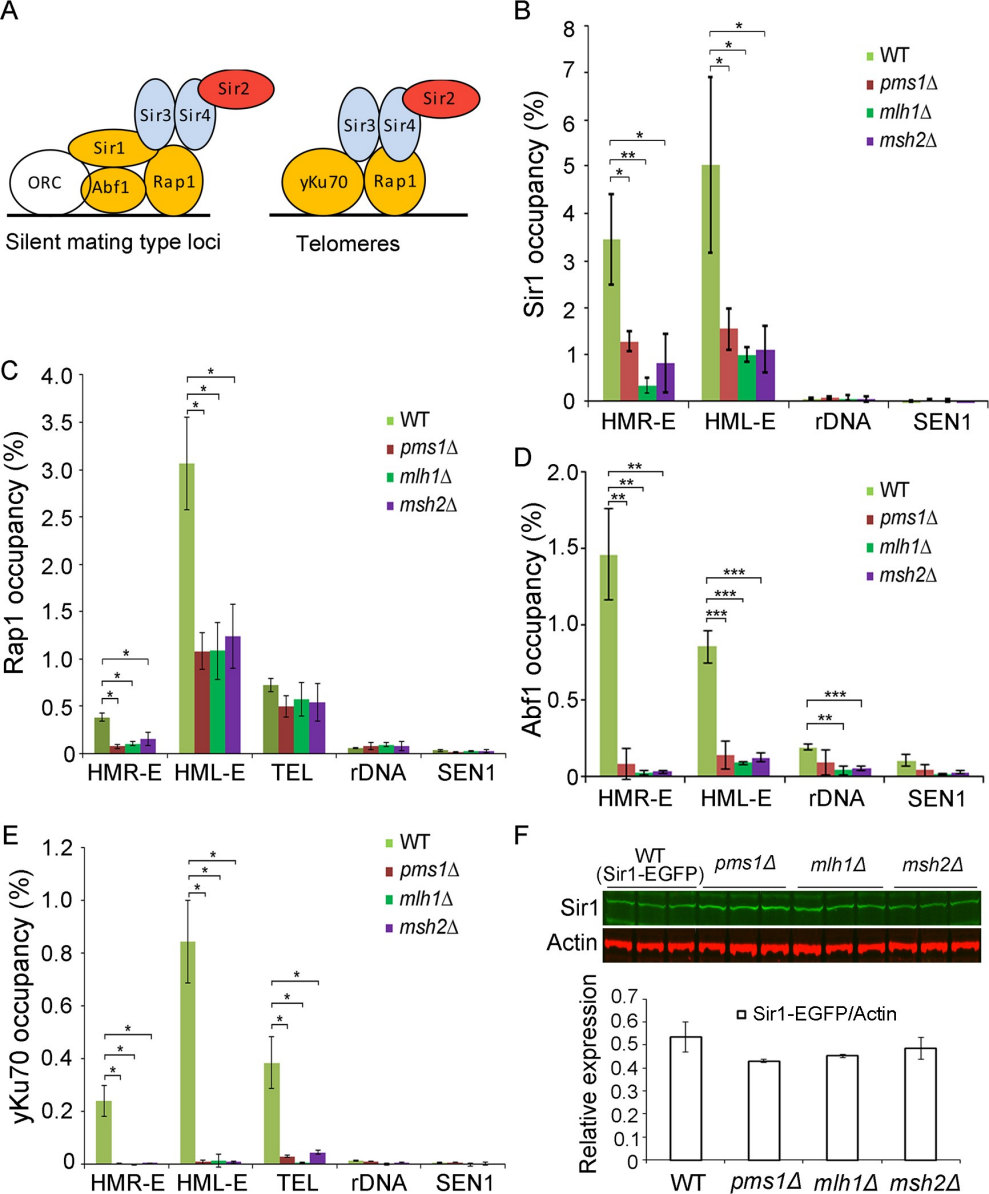
MMR deletions affect the occupancy of the silent complex recruiting components on the *HMR* and *HML* loci and telomeres. **(A)** Schematic showing the components of silent complex recruiting components (yellow) and the silent complex (Sir2, Sir3 and Sir4) at silent mating-type and telomere loci. **(B–E)** The occupancies of EGFP C terminal tagged Sir1, Rap1, Abf1, or yKu70 at mating-type, telomere, and rDNA regions were determined in the WT, *pms1*Δ, *mlh1*Δ, and *msh2*Δ. Experiments were performed in triplicate, and values are means ± SD. Statistical significance was determined using two-tailed Student’s *t*-tests; *, **, and *** indicate significance at *P* < 0.05, < 0.01, and < 0.001, respectively. **F**. Western blot analysis shows no significant changes in the expression level of Sir1-EGFP in the WT, *pms1*Δ, *mlh1*Δ, or *msh2*Δ using GFP antibody; actin was used as the loading control.

Silent-complex recruiting proteins Orc1, Abf1, Rap1, yKu70, and Sir1 initially bind to the silent mating-type loci or telomeres and then recruit the silent-complex Sir2, Sir4, and Sir3 to these loci. Orc1, Abf1, Rap1, and Sir1 are needed for establishing silencing at the *HMR* and *HML* loci, but Sir1 and Abf1 are not required for the establishment of silencing at telomere regions [38–41] (Fig 3A). Deficiency in the MMR process is known to cause accumulation of double-strand breaks in the cell [42–47]. Because the formation of double-strand breaks can cause Sir-recruiting proteins to be released from the silent loci, and because some of these proteins including yKu, Sir3, and Sir4 relocate to the damaged sites [48, 49], we determined whether MMR components influence silencing/chromatin structure by affecting the association of the silent-complex recruiting proteins. We first constructed the WT control strain and MMR deletion strains carrying the Sir1, Rap1, Abf1, or yKu70 proteins tagged with enhanced green fluorescent protein (EGFP). Primer positions corresponding to the *HMR-E*, *HML-E* locus, rDNA region, and the sub-telomeric region (*YFR057W*) are shown in S2G Fig. ChIP analysis indicated that Sir1, Rap1, Abf1, and yKu70 occupancies were significantly reduced at the silent mating-type loci in the MMR deletion strains (Fig 3B–3E). Previous reports showed that Rap1 and yKu70 are also enriched at telomeres, but that Sir1 and Abf1 are absent from telomere [50, 51]. We therefore checked the occupancy of Rap1 and yKu70 at the telomeres of the MMR mutant. No significant changes were observed in the occupancy of Rap1 at telomeres (Fig 3C), but the occupancy of yKu70 was significantly decreased at telomeres (Fig 3E). In the rDNA region, the occupancy level of all four proteins was very low, (Fig 3B–3E), which is consistent with the fact that the establishment of silencing at rDNA regions is controlled by another complex, the regulator of nucleolar silencing and telophase exit (RENT) complex [52–54]. Moreover, western blot analysis revealed no significant changes in the protein levels of the silent complex recruiting components (Sir1, Rap1, yKu70, and Abf1) in the MMR deletion strains, which ruled out the possibility that the observed occupancy changes were due to decreased protein levels (Fig 3F and S3 Fig). To exclude the potential effect of mutations on the binding sites of these proteins, we have also sequenced the DNA at mating type and sub-telomeric region in the MMR mutants. No rearrangements or mutations can be observed at the corresponding binding sites (S4 Fig). These results suggested that the deletion of MMR might lead to the disassociation of silent complex recruiting components from the *HMR* and *HML* loci and telomeres.

### Deletions of MMR components alter Sir2 localization and histone acetylation levels

**Fig 4:**
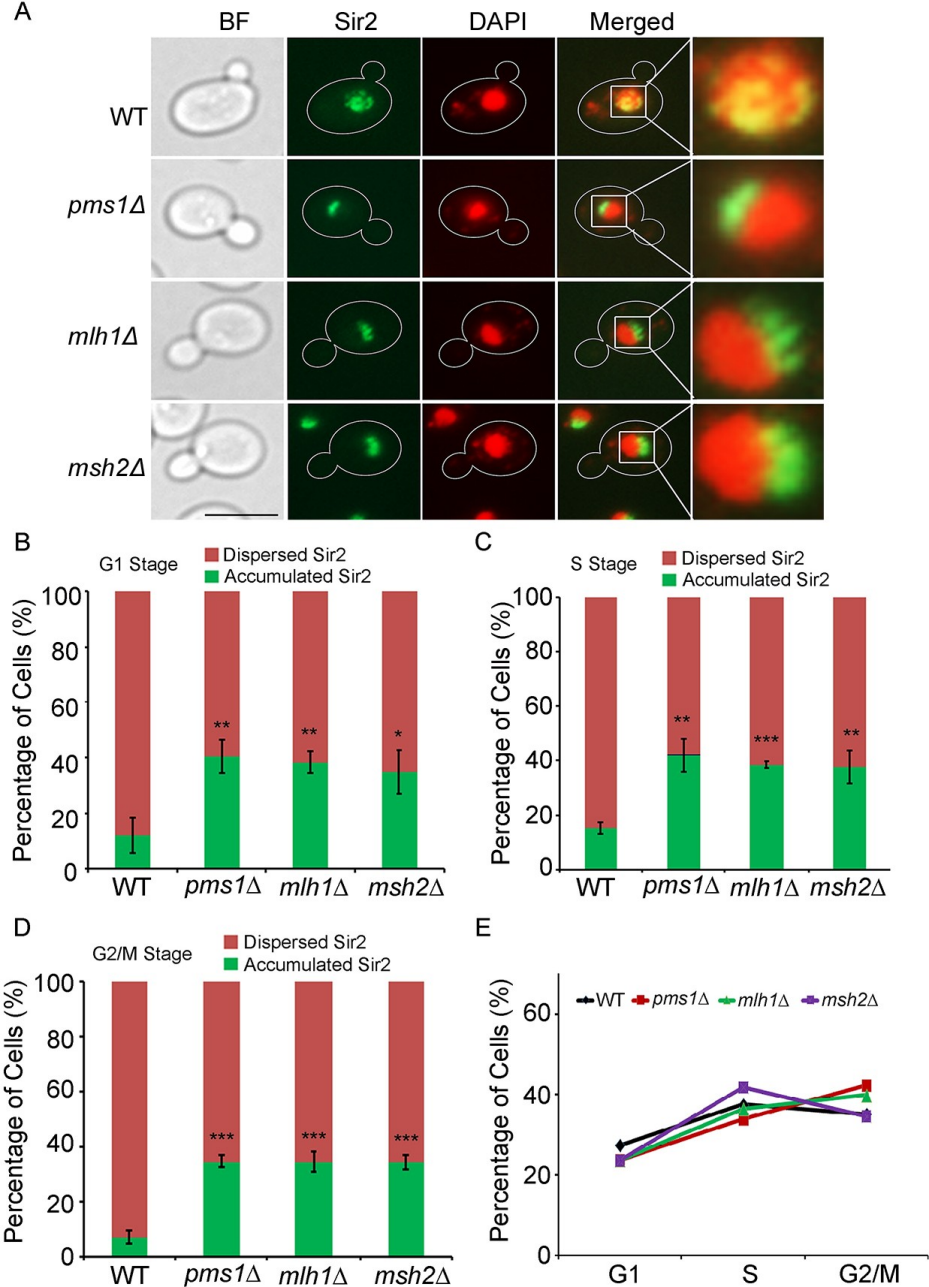
Deletion of MMR components alters the localization of Sir2 protein in the cell. **(A)** Conventional fluorescence microscopy shows that the Sir2 protein (green channel, labelled with Sir2-EGFP) is distributed throughout the nuclear region in the WT (Sir2-EGFP) but accumulates in a defined area in the nucleus (DAPI channel) in *pms1*Δ, *mlh1*Δ, and *msh2*Δ mutants carrying Sir2-EGFP. Scale bar = 5 μm. **(B)**, **(C),** and **(D)** The ratios of cells with accumulated Sir2 signal in the deletion mutants and WT were quantified in the **(B)** G1 stage, **(C)** S stage, and **(D)** G2/M stage. The red colour indicates the cells in which Sir2 proteins were dispersed—the Sir2-EGFP signal is distributed throughout the nuclear region as showed in the WT cell in (**A**), and the green colour indicates the cells in which Sir2 proteins were accumulated—the Sir2-EGFP signal accumulates in a defined area in the nucleus. Values are means ± SD. **(E)** The distribution of cells among cell cycle stages in WT and the MMR mutants. Experiments were performed in triplicate. A total 459 (Sir2-EGFP WT), 385 (*pms1*Δ), 549 (*mlh1*Δ), and 531 (*msh2*Δ) cells were counted. In **B**-**D**, statistical significance was determined using two-tailed Mann-Whitney U tests; *, **, and *** indicate significance at *P* < 0.05, < 0.01, and < 0.001, respectively.

**Fig 5:**
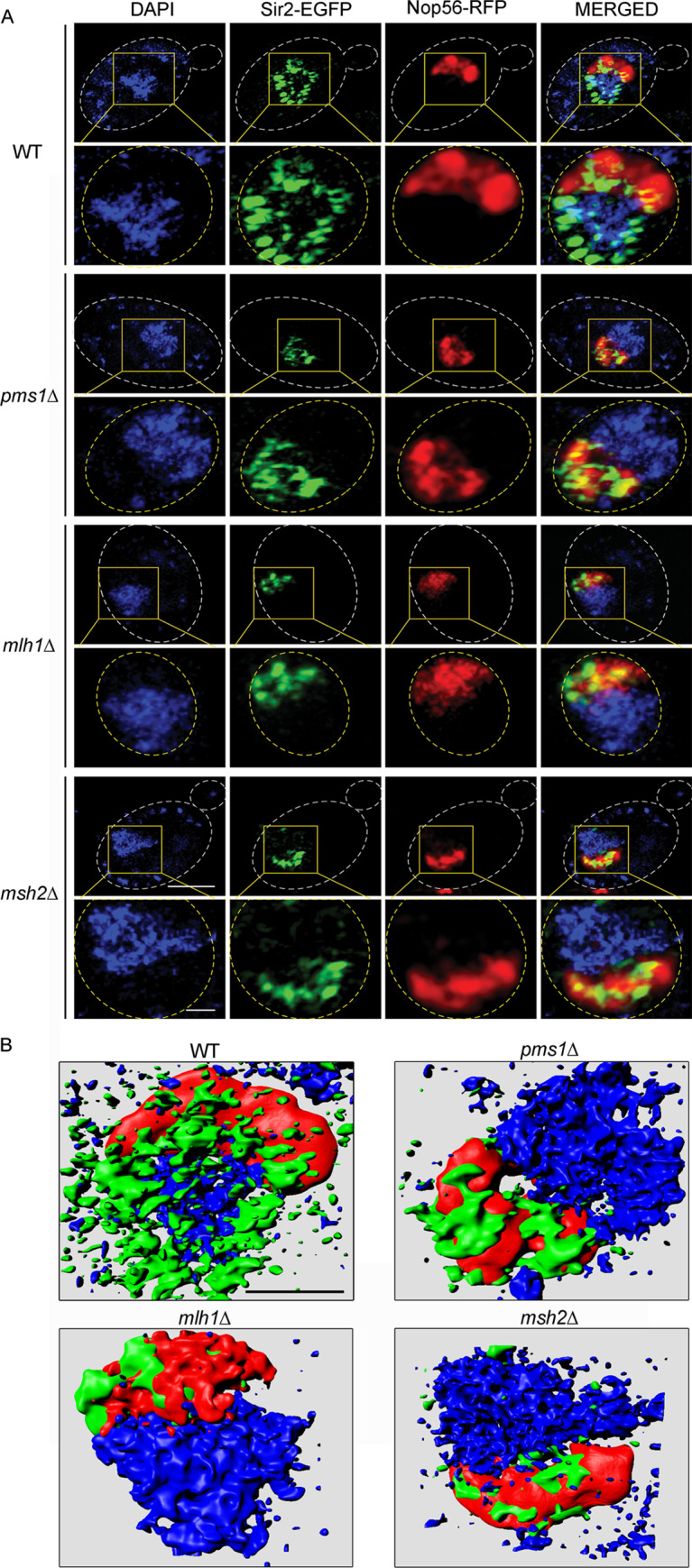
Sir2 co-localizes with the nucleolus marker Nop56 in MMR deletion mutants. **(A)** Sir2-EGFP co-localizes with Nop56-RFP in the MMR deletion mutants (*SIR2-EGFP pNop56-mRFP-LEU2 WT* and corresponded *pms1Δ*, *mlh1Δ*, and *msh2Δ* mutants) as revealed by super-resolution three-dimensional structured illumination microscopy (3D-SIM). Scale bar = 2 μm in the upper panel and 0.5 μm in the lower panel. **(B)** 3D-surface reconstructed images showing the subcellular localization of Sir2 (in green) in the indicated strains; fluorescence signals were reconstructed with 3D-surface using Imaris 7.2.3 software. Green, Sir2; Red, Nop56; Blue, DAPI. Scale bar is 0.5 μm.

The silent complex (Sir2, Sir4, and Sir3) is recruited by Orc1, Abf1, Rap1, yKu70, and Sir1 to the silent mating-type loci or by Rap1 and yKu70 to telomeres in the cell [38, 55]. We next wanted to determine whether such a disassociation of the silent complex recruiting components from the HMR and HML loci changed the distribution of the silent complex. To investigate this possibility, we assessed the subcellular localization of the key silent complex component Sir2 by using Sir2-EGFP as a marker. Intriguingly, we observed that the Sir2-EGFP signal, which is distributed throughout the nuclear region in WT cells [56], accumulated in a defined area within the nucleus in the mutants (Fig 4A). This observation suggested that Sir2 proteins might have disassociated from the mating-type and telomere loci in the MMR mutants, as occurs in the WT cells, but accumulated in other regions of the mutants. The association and distribution of SIR complex in the yeast cell depends on the cell cycle stage [57–59]. We then calculated the number of cells with accumulated Sir2 proteins at the G1, S, and G2/M stages (cells were grouped into cell cycle stages based on yeast cell morphology as previously described [60]), and the results demonstrated that the numbers of cells with accumulated Sir2 were independent of cell cycle stage and showed a significant increase at all cell cycle stages, i.e., at G1 (Fig 4B), S (Fig 4C), and G2/M (Fig 4D), in the MMR deletion mutants. To rule out the possibility that the observed Sir2 localization changes were caused by cell cycle arrest in the MMR mutants, we also quantified the number of cells at different cell cycle stages for WT and the MMR mutants. We found no significant differences between the WT and mutants (Fig 4E) in number of cells at different cell cycle stages, which indicated that the accumulated Sir2 proteins in the MMR mutants was not due to these mutations causing cells to be arrested at certain cell cycle stages. Previous studies reported that when other SIR proteins are absent (such as in *sir3Δ* or *sir4Δ*), Sir2 accumulates in the nucleolus/ rDNA regions [54, 61]. To investigate whether Sir2 accumulates in the nucleolus/rDNA regions in the MMR mutants, we performed a co-localization assay of Sir2 with a well-characterized nucleolus marker, Nop56 [62, 63]. Three-dimensional structure illumination microscopy (3D-SIM) clearly indicated that Sir2-EGFP co-localized with Nop56-RFP in the MMR deletion mutants (Fig 5A and 5B). 3D-SIM also revealed that Sir2-EGFP formed punctate foci that were distributed over the entire area of the nucleus (both the DAPI stained and Nop56-RFP regions) in WT cells. In the MMR deletion mutants, in contrast, Sir2-EGFP formed concentrated foci that were mainly localized in the nucleolus (Fig 5A and 5B and S1–S4 Movies). These observations indicate that MMR deficiency could cause Sir2 accumulation in the nucleolus/rDNA regions.

**Fig 6:**
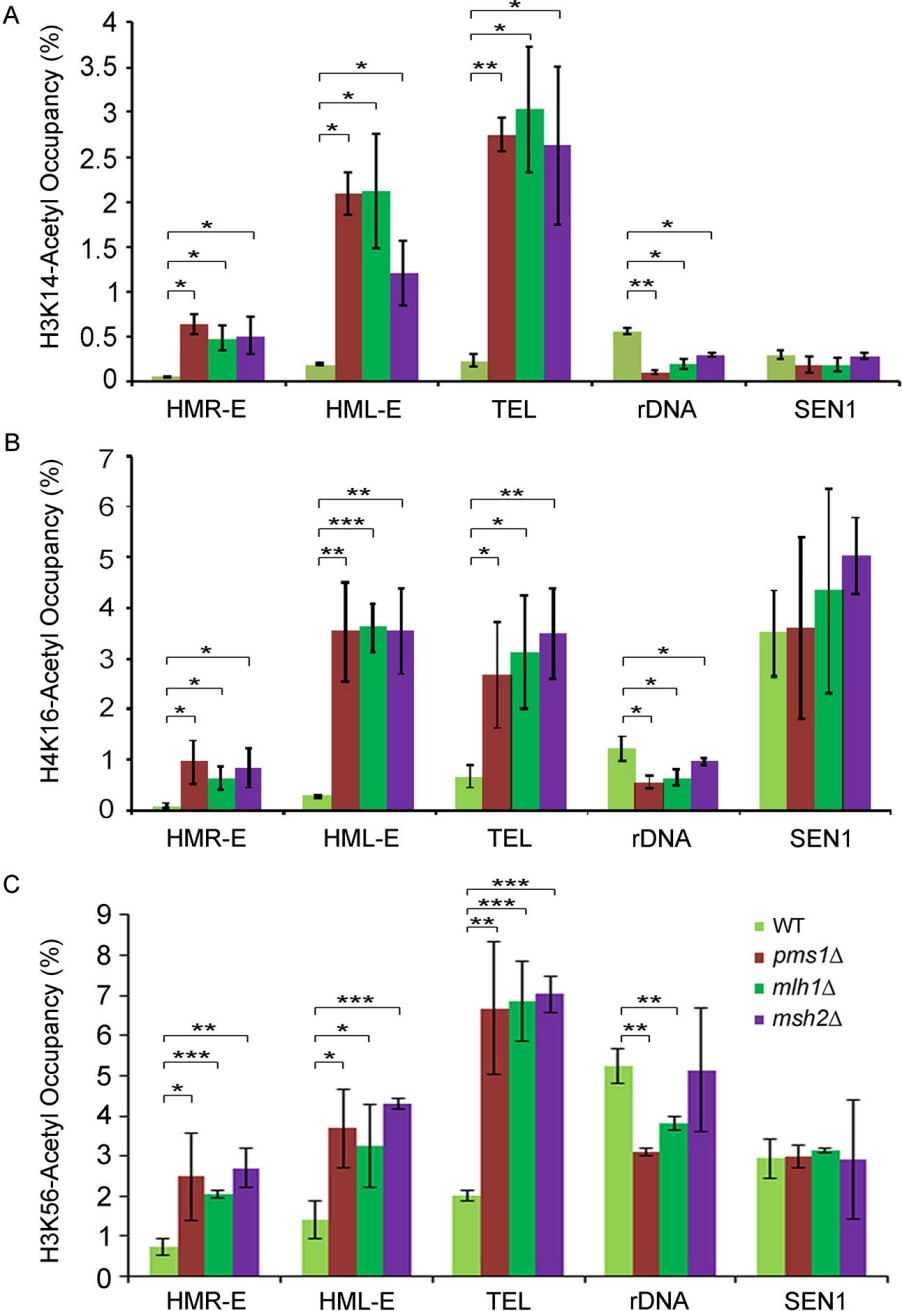
Deletion of MMR components alters histone acetylation levels at silent loci. The acetylation levels of H3K14 **(A)**, H4K16 **(B)**, and H3K56 **(C)** at mating-type silencer, telomere, and rDNA regions were assessed by ChIP-qPCR assay in WT (*his3Δ*), *pms1*Δ, *mlh1*Δ, and *msh2*Δ cells. In these deletion mutants, levels of H3K14, H4K16, and H3K56 acetylation were increased at the silent mating-type and telomere regions and were decreased in the rDNA regions (except for the H3K56 acetylation levels in the rDNA region of *msh2Δ*). Experiments were performed in triplicate, and values are means ± SD. Statistical significance was determined using two-tailed Student’s *t*-tests; *, **, and *** indicate significance at *P* < 0.05, < 0.01, and < 0.001, respectively.

Because Sir2a is a histone deacetylase, its disassociation from silent mating-type loci and telomeres in MMR mutants could potentially change patterns of histone acetylation at these loci. To investigate this possibility, we performed ChIP analysis in order to monitor three acetylation sites known to be targets of the Sir2 protein: H4K16 [64, 65], H3K56 [66, 67], and H3K14 [68]. In the MMR deletion mutants, levels of H3K14, H4K16, and H3K56 acetylation were significantly increased at the silent mating-type and telomere loci (Fig 6A, 6B and 6C), but were significantly decreased in the rDNA region (except for H3K56 acetylation levels in the *msh2Δ* mutants). Thus, these patterns of histone acetylation were consistent with the observed localization changes of Sir2 (Fig 5) and the silencing phenotype changes (Fig 2A) in the MMR mutants.

**Fig 7:**
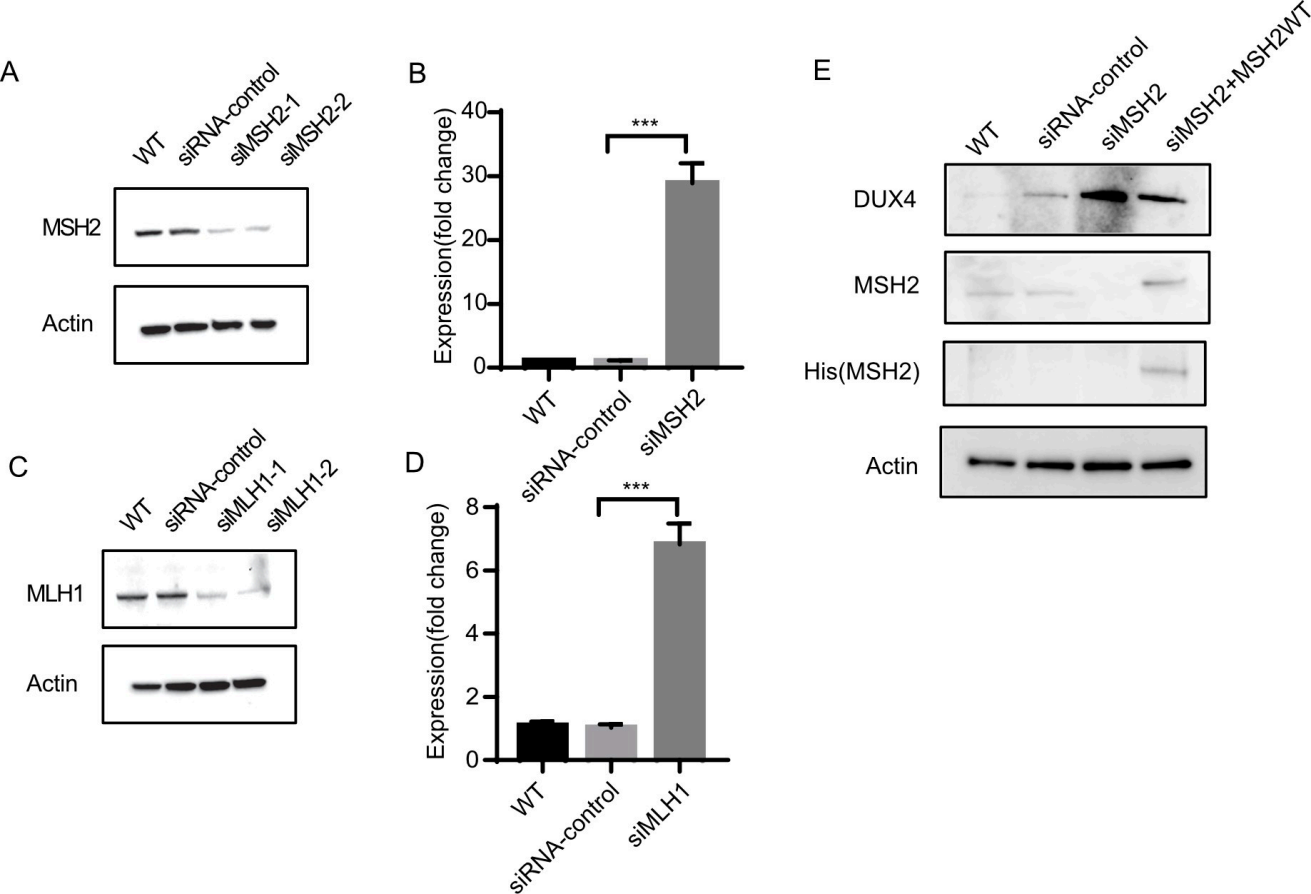
Knockdown of MMR components in HEK293T cells increases *DUX4* gene expression. (**A and C**) Western blots showing the protein level of MSH2 **(A)** and MLH1 **(C)** in siRNA-treated HEK293T cells. Non-treated (WT) and scrambled siRNA served as controls. Actin was used as a loading control. **(B** and **D)** RT-PCR analysis of *DUX4* gene expression in MSH2 **(B)** and MLH1 **(D)** knockdown cells. Experiments were performed in triplicate, and values are means ± SD. Statistical significance was determined using two-tailed Student’s *t*-tests. *** indicates significance at *P*<0.001. **(E)** Western blots showing the protein level of DUX4 in siRNA targeted to MSH2 treated HEK293T cells. siRNA resistant MSH2-His plasmid was transfected in siRNA treated cell simultaneously. The siRNA-mediated knock-down of endogenous MSH2 is shown by Western blotting using antibodies to MSH2 and the RGS-His-tag on the recombinant (siRNA-resistant) MSH2. Non-treated (WT) and scrambled siRNA served as controls. Actin was used as a loading control.

Finally, we determined whether depletion of MMR components affects gene silencing in mammalian cells. The expression of the double homeobox 4 (*DUX4*) gene, which is adjacent to the end of chromosome 4q, is regulated by telomere silencing [69]. We knocked down the expression of two MMR components, *MLH1* and *MSH2*, using siRNA in HEK293T cells (Fig 7A and 7C). The expression of full-length *DUX4* was significantly upregulated approximately 7 fold with the knockdown of *MLH1* and 28 fold with the knockdown of *MSH2*, respectively (Fig 7B and 7D). We also found that siRNA-mediated knockdown of endogenous MSH2 elvated DUX4 protein level (Fig 7E). Introduction of siRNA resistant, wild type MSH2 could restore MSH2 protein level and lead to reduced DUX4 protein expression (Fig 7E)[70]. Our results therefore demonstrated that MMR components are also involved in telomere-length related expression changes in human cells.

**Fig 8:**
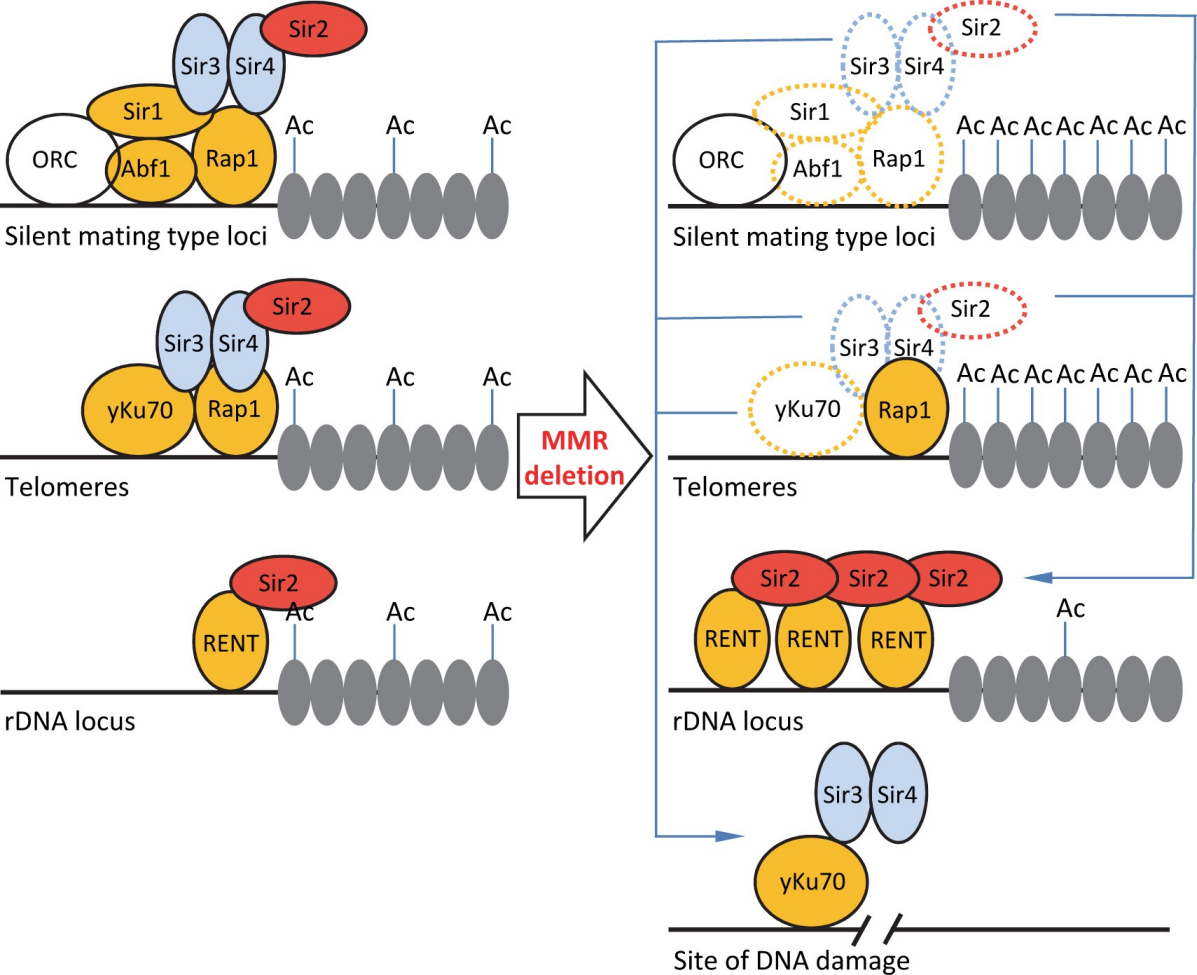
A hypothetical model of MMR-mediated epigenetic silencing. In WT cells (left panel), the silent complexes are normally established at the silent loci. Deletion of MMR components (right panel) significantly reduces the occupancy of Abf1, Rap1, yKu70, Sir1, and Sir2 proteins at the mating type and telomere silent regions, and lead toSir2 proteins are enriched at the rDNA regions; Accordingly, the acetylation levels are increased at the silent mating-type and telomere loci but decreased at rDNA regions. This potentially due to that yKu70, Sir3, and Sir4 are recruited to the increased DNA damage sites in the MMR mutants.

In summary, our study revealed that the mismatch repair components *MLH1*, *MSH2*, and *PMS1* are required for inheritance of gene silencing at silent mating-type loci and telomeres. Deletions of these genes caused the redistribution of the Sir2 deacetylase from silent mating-type loci and telomeres to rDNA regions. This increased acetylation levels at histone positions H3K14, H3K56, and H4K16 in silent mating-type loci and telomeres, but decreased acetylation levels in rDNA regions. These changes ultimately lead to the altered chromatin structures and silencing levels in the MMR mutants (Fig 8).

## Discussion

The main objectives of this study were to identify novel components involved in epigenetic silencing and to investigate the functional roles of these components in regulating epigenetic silencing. To achieve these objectives, we developed an SGA-based genome-wide silencing screen. Yeast SGA analysis has not been considered suitable for investigation of components affecting mating-type silencing, because mutants that affect mating-type silencing have normally lost the ability to mate. Proper mating ability is essential for the strain crossing required for homozygous diploid construction, which is the first step in the SGA method. This limitation hinders the isolation of deletion mutants, which lead to a complete loss of silencing at the mating-type loci. Fortunately, strong silencing regulators, such as yKu70, Sir2, Sir3, and Sir4, were previously identified from haploid sterility screens [13, 14]. Our work focused on identifying novel genes involved in epigenetic silencing at the *HMR* locus. We expected that the deletion of certain genes would cause a fraction of the cells to lose silencing at the locus tested but that the deletion strains would still retain their mating ability. It should be noted that this project only screened for components that alter the *HMR* silencing, the differences among the silencers of *HMR* from *HML* and telomeres may produce differing effects at the latter two loci.

The potential role of MMR components in the control of epigenetic silencing has not been specifically addressed before. But a previous genome-wide study found that deletion of one of the MMR components *MSH2* can enhance silencing defects of a yeast proliferating cell nuclear antigen mutant [71]. In line with this, we found that deletion of *MSH2* caused a significant decrease of silencing at the silent mating-type loci. These results indicate that Msh2 might have a role in controlling epigenetic silencing at the mating-type loci.

In *S*. *cerevisiae*, studies have shown that the passage of cells through S phase and a functional autonomous replication sequence (ARS) component of the *cis*-acting silencer element linked to the *HMRa* locus are essential for the establishment of silencing at the *HMRa* locus [72, 73]. Other researchers have also shown that mutations in the genes encoding the ORC subunits at the ARS elements causing mating-type silencing defect in *S*. *cerevisiae*[74–78]. Moreover, mutations affecting DNA polymerase and helicase components can disrupt silencing [79]. These results established a clear link between the DNA replication machinery and mating-type silencing in yeast. Moreover, MMR components physically interact with the DNA replication machinery [19] and co-localize with the DNA replication machinery during the S phase in a manner that is independent of the mismatched DNA repair function [80]. These previous data and our results seems to indicate that MMR components might potentially influence silencing during DNA replication process.

It is known that MMR mutants can cause the accumulation of double-strand breaks in the cell during DNA replication [42–46], which lead to further relocation of Sir3 and Sir4 from the silent mating-type loci and telomeres to the damaged sites [48, 49]. And Sir2 is found to accumulate in the nucleolus when Sir3 or Sir4 are absent [54, 61]. Our results extend these studies by showing that deletion of MMR components alters the occupancy of the silent complex recruiting proteins at the silent mating-type loci and telomeres. Deletion of these genes probably led to an open chromatin structure at silent mating-type cassettes and telomere loci and a more compact structure at rDNA loci. Moreover, our results indicated that such changes in chromatin structure in the MMR mutants were probably caused by an altered Sir2 localization and modified histone acetylation levels at these loci. Taking together these previous results with our data we generate a hypothetical model on how MMR components influence silencing. That is the absence of MMR components can lead to the accumulation of double-strand breaks [42–46], such accumulation could further lead to the relocation of yKu70, Sir3, and Sir4 from the silent mating-type and telomere loci to the DNA damage sites. This eventually causes the accumulation of Sir2 at the rDNA regions observed in the MMR mutants in our work; the Sir2 relocation and accumulation then changes the epigenetic silencing patterns at these loci. There are multiple ways could be used to further test this hypothetical model, first of all a γ-H2AX immunofluorescence microscopy analysis could provide evidence for increased levels of global DNA damage in the MMR mutant cells. Whether the DNA damage foci co-localize with any reduced silencing-complex recruiting proteins can be tested also by using this method. Moreover, a yKu70 ChIP-Seq assay to visualize the genome-wide patterns of yKu70 occupancy in the MMR mutants could be used for further verifying this hypothesis. Furthermore, it will be informative and to interesting test whether overexpression of the relocated factors such as yKu70, Sir3, and Sir4 (or even Sir2) might be enough to rescue the decreased silencing phenotype observed in the MMR mutants.

A possible explanation of the observed decreased Abf1 association in the MMR mutants could be due to that the MMR complex is also known to be involved in the correction of mismatches that arise during the formation of heteroduplex DNA between two homologous chromosomes during meiotic recombination [81]. Thus deletion of the MMR components could lead to an increased single strands of heteroduplexes, which may trigger nucleotide excision repair pathway [82]. Abf1 is a key component of the nucleotide excision repair pathway through interacting with Rad7-Rad16 complex[83, 84]. Thus more Abf1 could be potentially recruited to these single strand of heteroduplexes in the MMR mutants (Fig 8). MMR mutants lead to high rate of DNA mutations. It could be interesting to determine whether DNA mutations might also contribute to gene silencing status in the MMR mutants, which might provide a potential new oncogenesis mechanism. Thus future studies on characterizing the contribution of DNA mutations on gene silencing status may provide important insight into MMR oncogenesis.

The MMR components identified in this study are highly conserved from yeast to human. Mutations in MMR components have been associated with cancer development in humans. MMR deficiency leads to microsatellite instabilities (MSI), which are observed in most cancers, including colorectal, uterine, stomach, ovary, and small intestine cancers [85–88]. Researchers have found a perfect association between MMR immune-histochemical analysis and MSI in most cases of hereditary nonpolyposis colorectal cancer (HNPCC) and in 15–20% of cases of sporadic colorectal cancer [89, 90]. Furthermore, mutations in Mlh1 can affect MMR tumour suppressor functions in a tissue-specific manner [22]. The increased expression level of *Dux4* has been confirmed as one causal factor of facioscapulohumeral muscular dystrophy (FSHD)[91–93], which is one of the most prevalent myopathies. The elevated expression of full-length *DUX4* produces muscle toxicity and leads to cell death. Our results suggest the MMR component depletion or mutations leads to dysregulated gene silencing and may thereby contribute to disease pathogenesis. Indeed, correlations between FSHD and cancers have been reported [94, 95]. These and other results have established a strong connection between deficient MMR and cancer development. The current findings strengthen the evidence for correlation by revealing that MMR components function in regulating epigenetic silencing. Our results may also prove relevant for understanding the mechanism of oncogenesis caused by MMR deficiency. Further evaluation of the silencing regulation effects of the MMR components in mammalian model systems may provide more direct evidence of their role in epigenetic silencing regulation.

In summary, our study has revealed that MMR proteins are required for epigenetic silencing at mating-type and telomere loci. Deletions of *PMS1*, *MLH1*, and *MSH2* altered chromatin structure. Interestingly, we also found that Sir2 protein accumulated at rDNA regions in the MMR deletion mutants; this accumulation increased histone acetylation levels at mating-type and telomere loci and decreased histone acetylation at the rDNA loci. We identified a novel link between MMR and epigenetic silencing (Fig 8). Further studies of regulation of epigenetic silencing by these MMR components in relevant cancer patient samples will increase our understanding of MMR-related oncogenesis.

## Materials and methods

### Strains, plasmids, and primers

The yeast strains, plasmids, and primers used in this study are listed in S1 and S2 Tables. All yeast strains are in BY4741 background, except for the strains (UCC3505) that were used for telomere silencing assays. Since we do not have a BY4741 strain that carrying the telomere silencing markers, we acquired the UCC3505 strain from Prof. Daniel Gottschling lab for the silencing assays at telomere locus. The standard lithium acetate/PEG method was used for yeast transformation, and constructed strains were verified by PCR and immunoblotting analysis. Yeast single gene deletion collection is a kindly gift from Prof. Charlie Boone.

### Genome-wide silencing screening procedure

The developed screening approach was generally based on the standard SGA approach [16]. The major difference is that, instead of working with markers that are normally expressed, this screen works with markers inserted into the silent mating-type loci, in which genes are supposed to be highly silenced. Thus, nicotinamide was added to the medium to remove the silencing effect at those steps that required the selection of progeny that harbor these markers at the silent mating-type loci.

The genome-wide silencing screen crossing procedure was performed in seven steps as follows: **1.** A *MATα* query strain Ywrl13 was crossed to the yeast single-deletion collection SGA*-*v2. **2.** Heterozygous diploids was selected on Ura dropout medium containing hygromycin B and G418 with nicotinamide (5 mM). **3.** Sporulation was induced using standard SGA sporulation medium. **4 & 5.** *MATa* haploid and Kan selection. **6.** Final triple-selection (-Ura, + G418, + hygromycin B and +nicotinamide (5 mM)). **7.** HMR locus silencing phenotype measurement. See below for detailed information regarding each steps (All the pinning steps were performed using a Singer RoTor HAD pinning robot (Singer Instruments)).

1. **Mating:** A *MATα* query strain Ywrl13 carrying *hmra1-a2Δ*::*URA3* and *hmlα1-α2Δ*::*HphR* (See S1 Table for genotype details) was used. The query strain was constructed from previous work in our group [24]. This query strain was crossed to an ordered array of *MATa* yeast single-deletion mutants (SGA-v2) each carrying a gene deletion with a *kanMX4* marker. The mating was performed on Yeast extract-peptone-dextrose growth medium (YEPD) using the pinning robot. Plates were then incubated at 22°C for 1 day.
2. **Diploid selection:** To select for diploids, pin the resulting MAT a /α diploid zygotes to diploid selection medium (described below and in the Genome-wide silencing screening media section). Incubate at 30°C, 2 days. Growth of resultant heterozygous diploids was selected on Ura dropout medium containing hygromycin B and G418 together with nicotinamide (5 mM), which inhibits the enzymatic activity of Sir2 and releases the repression of the *URA3* and *HphR* markers at the silent mating-type loci.
3. **Sporulation:** Heterozygous diploids were transferred to an SGA sporulation medium with reduced levels of carbon and nitrogen to induce sporulation. Incubate at 22°C, 7 days.
4. ***MATa* meiotic progeny selection:** Spores were transferred to a standard SGA haploid selection medium lacking histidine, arginine, and lysine, and containing canavanine and thialysine for *MATa* haploid selection. Incubate at 30°C, 2 days. Canavanine is a toxic analog for arginine and thialysine is a toxic analog for lysine. The query strain carrys *can1Δ* and *lyp1Δ*, this means that *MATa/alpha* diploid cells can be killed by canavanine and thialysine because they carry a wild-type copy of the *CAN1* and *LYP1* genes.
5. ***MATa* kanR selection:** The *MATa* meiotic progeny were then transferred to a Kan selection medium (haploid selection medium + G418). Incubate at 30°C, 1–2 days. This step only aims to select *MATa* haploid cells carrying the single-gene deletions.
6. **Final triple selection:** The haploid *MATa* single deletions carrying the silencing markers *URA3* and *HphR* were selected on a final triple-selection medium, a medium lacking uracil but containing G418, hygromycin B, and nicotinamide (5 mM). Plates were incubated at 30°C for 2 days. The nicotinamide was used here again to switch on the expression of the *URA3* and *HphR* markers at the silent mating-type loci and enable the growth of cells carrying these markers on the selection medium.
7. ***HMR* locus silencing phenotype measurement:** After acquisition of cells carrying the single deletions together with *URA3* and *HphR* markers at the silent mating-type loci in the previous step, only the silencing phenotype at the *HMR* locus was chosen as a read out for this screen (the expression of *HphR* at the *HML* locus was not measured). The strains were transferred onto SD medium lacking uracil. To bring the cells back to their normal silent state from the previous triple-selection plates, nicotinamide was not added to the medium for this step. As a consequence, most of the strains (including WT cells or any mutants that do not influence the silencing at the *HMR* locus) could not grow on this Ura dropout medium. This resulted in a non-growth phenotype on the selection medium for these strains due to the silencing of the *URA* marker at the locus as shown in Fig 1B (WT panel and the colonies around *pms1Δ*, *msh2Δ*, *mlh1Δ*, and *sir1Δ* mutants). This selection can reveal gene mutations that lead to a loss of silencing at the *HMR* locus, as indicated by a better growth phenotype as shown in Fig 1B (panels showing *pms1Δ*, *msh2Δ*, *mlh1Δ*, and *sir1Δ* mutants, the four colonies in the middle that showed growth on the Ura dropout medium). For the screen, the growth of all mutants (measured as colony size) from the deletion collection on the Ura dropout medium was compared with a set of plates containing only WT cells (which cannot grow on the Ura dropout medium and appear as small colonies that resulted from the small number of cells transferred from the previous selection plates). Mutants with larger colonies than the WT were considered candidates for lost silencing. The screen was performed in duplicate in a 1536-spot format, and every deletion strain was represented in quadruplicate on each plate. The selection plates were incubated at 30°C for 2 days for each step, except that the plates were incubated at 22°C for 7 days for the sporulation step.

### Genome-wide silencing screening media

1. Mating: Yeast extract-peptone-dextrose growth medium (YEPD)
2. Diploid selection: Synthetic Defined (SD)—Ura + G418 + hygromycin B + nicotinamide (5 mM)
3. Sporulation: SGA enriched sporulation medium [16]
4. *MATa* meiotic progeny selection: SD—His/Arg/Lys + canavanine + thialysine
5. *MATa* kanR selection: SD—His/Arg/Lys +canavanine + thialysine + G418
6. Final triple selection: SD—His/Arg/Lys/Ura +canavanine + thialysine + G418 + hygromycin B + nicotinamide (5 mM)
7. *HMR* locus silencing phenotype measurement: SD—Ura

### Scoring of silencing screening using SGAtools software

For scoring of statistically significant changes in the silencing phenotype at the *HMR* locus on plates, we used a web-based analysis system: SGAtools (http://sgatools.ccbr.utoronto.ca). SGAtools provides a platform that can automatically quantify colony sizes from images of agar plates, correct for systematic biases, and calculate a growth score relative to the colony sizes from a control set of plates [96]. The steps are as follows: 1, Plates were imaged with a regular digital camera and images were uploaded onto the SGAtools website. 2, Colonies were isolated based on the signal intensity difference between the colonies and the plate background. 3, Size of the colonies were measured and normalized. 4, The size difference was scored based on statistical analysis of the values generated from the four colonies for each strain.

### Bioscreen assay for hits confirmation

After the scoring, potential hits that had the highest statistical probability of being true silencing modifiers were tested by a Bioscreen assay. The cells of mutants that had a significantly increased colony size on the SD-Ura medium were transferred to SD-Ura liquid medium in a honeycomb microplate pre-culture at 30°C (without shaking) for 2 days, and 5 μL of cells will transferred into a new honeycomb microplate with 345 μL of fresh SD-Ura liquid medium. Then the growth rate of each mutant was measured using a Bioscreen C mini-liquid culture machine (Oy Growth Curves AB) [24]. The optical density (OD600) was measured every 30 minutes for 48 hours at 30°C with shaking. Data was processed using the Excel program (Microsoft Office). Experiments were performed in triplicate, and statistical analysis was performed using unpaired two-tailed Student's *t*-tests by comparing growth rates between mutants and WT cells in SD-Ura medium.

### Yeast spot test

Yeast spot tests were performed and analysed according to the standard protocol on SD-Ura medium. For the adenine (red pigment formation) assay the telomere loci strains (UCC3505) and corresponding MMR mutants were spotted (OD 0.6 in first column) onto YPD plates and incubated for 2 days at 30°C followed by storage in 4°C until clear red pigment formation could be seen (15 days)[97]. Mating-type URA3 reporter strain (WT (*his3Δmsh2*Δ)) and telomere URA3 reporter strain (UCC3505 *msh2*Δ) were transformed with two plasmids (*pRS425* and *pRS425-GAL10-MSH2*). The *GAL10-MSH2* fragment was digested (BamHI and KpnI) from *pEAE86* (*GAL10-MSH2 2μm TRP1*) plasmid [98], a kind gift from Prof. E. Alani. Cells were precultured in SD-LEU + 2% Raffinose media before being serially diluted and spotted (OD 0.6 in the first column) onto SD-ura-leu + 2% glucose or SD-ura-leu + 2% galactose agar plates. The plates were incubated at 30°C for 2–3 days.

### Functional enrichment analysis

The functional enrichment analysis was performed using Gene Ontology Term Finder [99]. The list of confirmed hits from the spot test assay were uploaded on the Gene Ontology Term Finder website (https://www.yeastgenome.org/goTermFinder). The background list of the SGA-V2 array (contains 4261 genes) was also uploaded. The hits were analysed for the enrichment of GO bio-processes groups by comparison with the background list; Three ontology groups were analysed respectively, they are biological process, molecular function, and cellular component ontologies (see Boyle et al (2004) [99] for detailed information regarding these groups). P-values were calculated by using a hypergeometric distribution with multiple hypothesis correction, and the cut-off was set to P < 0.01.

### Interaction network analysis

The physical interaction network diagrams were extracted from the interaction analysis by using Ospery 1. 2.0 [100]. First, the confirmed hits list from the spot test was used as an input gene list. And then the software extracted the physical interactions between these hits from the BioGRID interaction database [27]. The software represented genes as nodes and interactions as edges between nodes. Lastly, the network layout was adjusted manually and images of the network was exported in portable network graphics (PNG), and scalable vector graphics (SVG) format for final processing.

### qRT-PCR

After the yeast cells were cultured to an OD_600_ value of 0.6 to 0.8, they were centrifuged for 5 min at 3000 rpm. The pellets were washed twice in cold phosphate-buffered saline (PBS) and re-suspended in 400 μl of TRIzol reagent (Ambion). The suspended cells were lysed with 400 μl of glass beads (Sigma-Aldrich) in a FastPrep-24 (MP Biomedicals) machine (run 20” stop 1 min for 5 cycles with power 6.5). The yeast cell lysates were collected in the Eppendorf tube and followed by the Chloroform:isoamyl-alcohol (25:1) extraction. RNA was precipitated with sodium acetate (NaAc 3M, pH 5.2) and ethanol (99%, -20°C), left at -20°C for at least 2 h, and centrifuged for 20 min at 15000 rpm at 4°C. RNA pellets were washed with cold 75% ethanol and then dissolved in 60 μl of sterile Rnase-free water. RNA samples were treated with RNase-free DNase I (New England BioLabs, M0303S), and cDNAs were made with the iScript cDNA Synthesis Kit (BIO-RAD,170–8890). Real-time qPCR was performed on a CFX96 Real-time system using iQ SYBR Green supermix (BIO-RAD).

### Mating-type silencing assay

Identified strains were picked up from the Final-Ura selection plates and re-streaked on SD-Ura plates. Colonies for each strain were picked from the latter plates, and spot tests were performed based on the standard protocol on SD-Ura media using 5-fold or 10-fold dilutions.

### Fluorescence microscopy

Fluorescence imaging was performed with a Carl Zeiss axiovert 200M wide-field fluorescence microscope with a 100× (NA = 1.4), oil, plan apochromatic correction Zeiss objective. Image quantification was performed using Image J (https://imagej.nih.gov/ij/index.html). For quantification of the Sir2 subnuclear localization in each cell cycle phase, cells were grouped into different cell cycle stages based on their morphology as previously described [60]. Experiments were performed in triplicate, and a total of 385–549 cells were counted for each sample.

### Chromatin immunoprecipitation (ChIP) assays

ChIP assays were performed as described previously [101]. Mid-log phase yeast cells were crosslinked by 1% paraformaldehyde, and 0.125 M glycine was used to quench fixation. Lysis buffer (50 mM Hepes-KOH, pH 7.5, 0.1% SDS, 1% Triton X-100, 0.1% sodium deoxycholate, 1 mM EDTA, 150 mM NaCl, and protease inhibitor) and glass beads were used to break the cells on a FastPrep-24 machine (ZYMO RESEARCH). After sonication and centrifugation, supernatants containing 25 μg DNA were incubated with 1 μg of antibodies or without antibody overnight at 4°C. Protein A beads were added, and the samples were incubated for 2 h. After washing and eluting steps, samples were incubated at 65°C overnight to reverse cross-link. RNA contaminants were removed by treatment with 0.2 μl of 20 mg/ml RNase A for 30 min at room temperature. Proteins were removed by treatment with 20 μg of Proteinase K for 2 h at 55°C. DNA was purified with the ChIP DNA Clean and Concentrator Kit (ZYMO RESEARCH). The purified DNA was used for real-time PCR analysis (Bio-Rad). Quantifications were performed using real-time PCR software (Bio-Rad) and Excel (Microsoft); the enrichment values were normalized to the input DNA values. SEN1 primers were used as a negative control in ChIP-qPCR experiments [102]. GFP antibody (ab290), H3K56ac antibody (ab71956), H3K14ac antibody (ab52946), and H4K16ac antibody (ab109463) were acquired from Abcam.

### Immunoblotting

Freshly prepared yeast cells were re-suspended in 0.2 M NaOH and incubated on ice for 20 min. After a brief centrifugation, the pellets were re-suspended in HU buffer (200 mM phosphate buffer, pH 6.8, 8 M urea, 5% SDS, 1 mM EDTA, bromophenol blue, and 1% β-mercaptoethanol) and were incubated at 70°C for 10 min. After centrifugation for 5 min at 11,200 *g*, a 10-μl volume of each supernatant was subjected to Bis-Tris protein gels (NuPAGE, NP0326, ThermoFisher SCIENTIFIC) electrophoresis, and the transferred nitrocellulose membranes were immunoblotted with GFP primary antibody (ab290, Abcam) and β-Actin primary antibody (ab8227, Abcam). HEK293T cells were lysed in cell lysis buffer (50 mMTris-Cl, pH 7.5, 150 mM NaCl, 1% Triton X-100 and 1x protease inhibitors (Roche)). Protein concentration was measured using the BCA assay (Thermo Scientific), and equal amounts of proteins were diluted in SDS-sample buffer (Bio-Rad), heated to 95°C for 5 min before SDS-PAGE. The transferred nitrocellulose membranes were immunoblotted with MSH2 (Santa cruz, sc-376384) or MLH1 (Santa cruz, sc-271978) and Actin (ab8227, Abcam).

### Super-resolution three-dimensional structured illumination microscopy (3D-SIM)

WT and mutant cells carrying Sir2-EGFP and Nop56-mRFP were incubated in SD-Ura-Leu medium at 30°C overnight. Cells were diluted in the same selective medium to an OD_600_ value of 0.2 and were then incubated at 30°C until the OD_600_ value increased to 0.5. The cells were then fixed with 3.7% formaldehyde and washed three times with PBS (pH 7.4) before they were stained for 1 h with 1 μg/ml of 4',6-diamidino-2-phenylindole (DAPI) (Sigma-Aldrich) in the dark and at room temperature. The cells were again washed three times with PBS (pH 7.4). 3D-SIM was performed using previously described settings[103]. Excitation light wavelengths of 405 nm (DAPI), 488 nm (GFP), and 561 nm (mRFP) were used. Image acquisition, super-resolution processing, and calculation were performed with ZEN black2-1SP1 (Carl Zeiss, Jena Germany). 3D images were reconstructed and animated using Imaris 7.2.3 software (Bitplane, Zurich, Switzerland).

### siRNA knockdown

About 200,000 HEK293T cells were plated in each well of a 6-well plate. After 24 h, siRNAs (20 nM, Qiagen) were transfected into the cells using Lipofectamine RNAiMAX (Invitrogen, 13778075) according to the manufacturer’s instructions. Cells transfected with scramble-control siRNA (Qiagen, 1022076) were used as controls. Cells were harvested at 72 h after transfection for RNA and protein analysis. The sequences of the siRNA target MLH1 were GTGGCTCATGTTACTATTACA and AACCATCGTCTGGTAGAATCA. The sequences for the siRNA target MSH2 were TCCAGGCATGCTTGTGT TGAA and CCCATGGGCTATCAACTTAAT.

Hela cells were reverse transfected with 20 nM On-target Smart Pool siRNA to MSH2 (Dharmacon) using Lipofectamine RNAiMAX (Invitrogen). Plasmid DNA(pcDNA-DEST40-RGSHis-MSH2)[70] were transfected with FugeneHD (Promega) at a DNA:FugeneHD ratio of 1:3, according to the manufacturer’s instructions. For transfection in 6-well plates, 1 μg of plasmid DNA per well was used. The mix of siRNA and plasmid was then added to newly-seeded cells and medium replaced with complete DMEM after 24 hours. Experiments were performed at 72 hours after transfection.

### DNA sequencing

Genomic DNA was extracted from one clone per strain using the MasterPure Yeast DNA Purification Kit from Epicentre. The region of interest was PCR amplified and sent to Eurofins Genomics for Sanger sequencing. The primers used for sequencing can be found in S2 Table. The Align Multiple DNA Sequences tool in SnapGene was used for displaying sequence alignments.

### Statistical analysis

All experiments were performed three times, and values shown are means ± SD of three replicates. Differences between means were assessed with unpaired two-tailed Student’s *t*-tests; *, **, and *** indicate statistical significance at *P* < 0.05, < 0.01, and < 0.001, respectively. For analysis of data in Fig 4E, a two-tailed Mann-Whitney U test was used.

## Supporting information

S1 Fig A flow diagram of the overall silencing screening procedure (A) and Spot tests confirmed the decreased mating-type silencing phenotypes of the deletion mutants identified from the silencing screen (B). Cells were 10-fold serially diluted and then spotted onto SC (left) and SD-Ura (right) agar plates; the *sir1Δ* mutant served as a positive control.
(TIF)

S2 Fig MMR mutants show decreased mating-type silencing. **(A)** MMR mutants have increased growth rates in SD-Ura liquid medium. The growth rates were measured using the Bioscreen mini-liquid culture approach. **(B)** Confirmation of *pms1Δ*, *mlh1Δ*, and *msh2Δ* decreased mating-type silencing phenotype by PCR knockout using the *natMX4* marker. The *sir1Δ* mutant obtained from the silencing screen was used as a positive control. **(C)** Deletion of MMR component gene *MSH6* didn’t affect mating type silencing. *sir1Δ* mutant was used as a positive control. **(D)** Cells of indicated strains were 10-fold serially diluted and then spotted onto SC (left), SD-Ura (middle) and SC+FOA(right) agar plates, the *sir1Δ* mutant served as a positive control. **(E)** Loss of silencing at telomere *ADE2* reporter visualized by red color formation. WT (UCC3505) and the corresponding MMR mutants were grown on YPD medium. MMR deletion strains (*pms1Δ*, *mlh1Δ*, and *msh2Δ*) displayed white with sectors colonies as compared to WT, indicating a loss of gene silencing at the telomere *ADE2* reporter. Cells were five-fold serially diluted and grown at 30℃ followed by storage in 4℃ until clear red pigment formation could be seen (15 days). **(F**) Decreased telomere silencing in the *MSH2* mutants were rescued by overexpressed *MSH2*. The plasmids (*pRS425* and *pRS425-Gal10-MSH2*) were transformed to *msh2*Δ mutants in both WT (*his3Δ*) and UCC3505 backgrounds. Cells were five-fold serially diluted and then spotted onto SD-ura-leu + 2% glucose (left) or SD-ura-leu + 2% galactose (right) agar plates. Overexpressed *MSH2* (OE *MSH2*) in the *msh2*Δ mutants partially restore telomere and mating type silencing, as compared to overexpressed empty vector (EV). **(G)** Schematic diagram showing the position of primers corresponding to: *HMR* (*HMR-E)* and *HML* (*HML-E*) loci on chromosome III, the rDNA (*NTS1/2*) on chromosome VII, and the TEL (*YFR057W*) on the right arm of chromosome VI. These primers were used in chromatin immunoprecipitation (ChIP) experiments and gene expression (*YFR057W*).
(TIF)

S3 Fig Western blot analysis using GFP antibody revealed no significant changes in the protein levels of Rap1 **(A)**, yKu70 **(B)**, or Abf1 **(C)** in the MMR deletion mutants compared to the WT.
(TIF)

S4 Fig Sequence alignments display no mutations in the regions of interest for the MMR mutants. Multiple sequence alignments of the MMR mutants (pms1Δ, msh2Δ and mlh1Δ) compared to WT strain using the Align Multiple DNA Sequences tool in SnapGene. A. Sequencing results of the binding sites of Abf1 (top) and Rap1 (bottom) in the HML locus. B. Sequencing results of the HMR locus with the binding sites of Rap1 and Abf1 highlighted. C. Sequencing results of the Tel6R region. Yellow color indicates matching bases.
(TIF)

S1 Table Strains used in this study. (XLSX)

S2 Table Primers used in this study. (XLSX)

S3 Table Candidate list generated from the genome-wide silencing screen of mutants showing a decreased silencing phenotype. (XLSX)

S4 Table Bioscreen mini-liquid culture approach confirmed the decreased silencing phenotype of mutants isolated from the genome-wide silencing screening. (XLSX)

S5 Table Drop test confirmed mutants with decreased silencing phenotypes. (XLSX)

S1 Movie 3D-SIM revealed that Sir2-EGFP formed punctate foci that were distributed over the entire area of the nucleus in WT cells. (MOV)

S2 Movie 3D-SIM revealed that Sir2-EGFP formed foci mostly localized in the nucleolus in *PMS1* deletion mutants. (MOV)

S3 Movie 3D-SIM revealed that Sir2-EGFP formed foci mostly localized in the nucleolus in *MLH1* deletion mutants. (MOV)

S4 Movie 3D-SIM revealed that Sir2-EGFP formed foci mostly localized in the nucleolus in *MSH2* deletion mutants. (MOV)
