# Supplementary figures and images for: Yeast mismatch repair components are required for stable inheritance of gene silencing
Source: PLoS Genet. 2020 May 29;16(5):e1008798. doi: 10.1371/journal.pgen.1008798 (PMC7286534; doi:10.1371/journal.pgen.1008798)

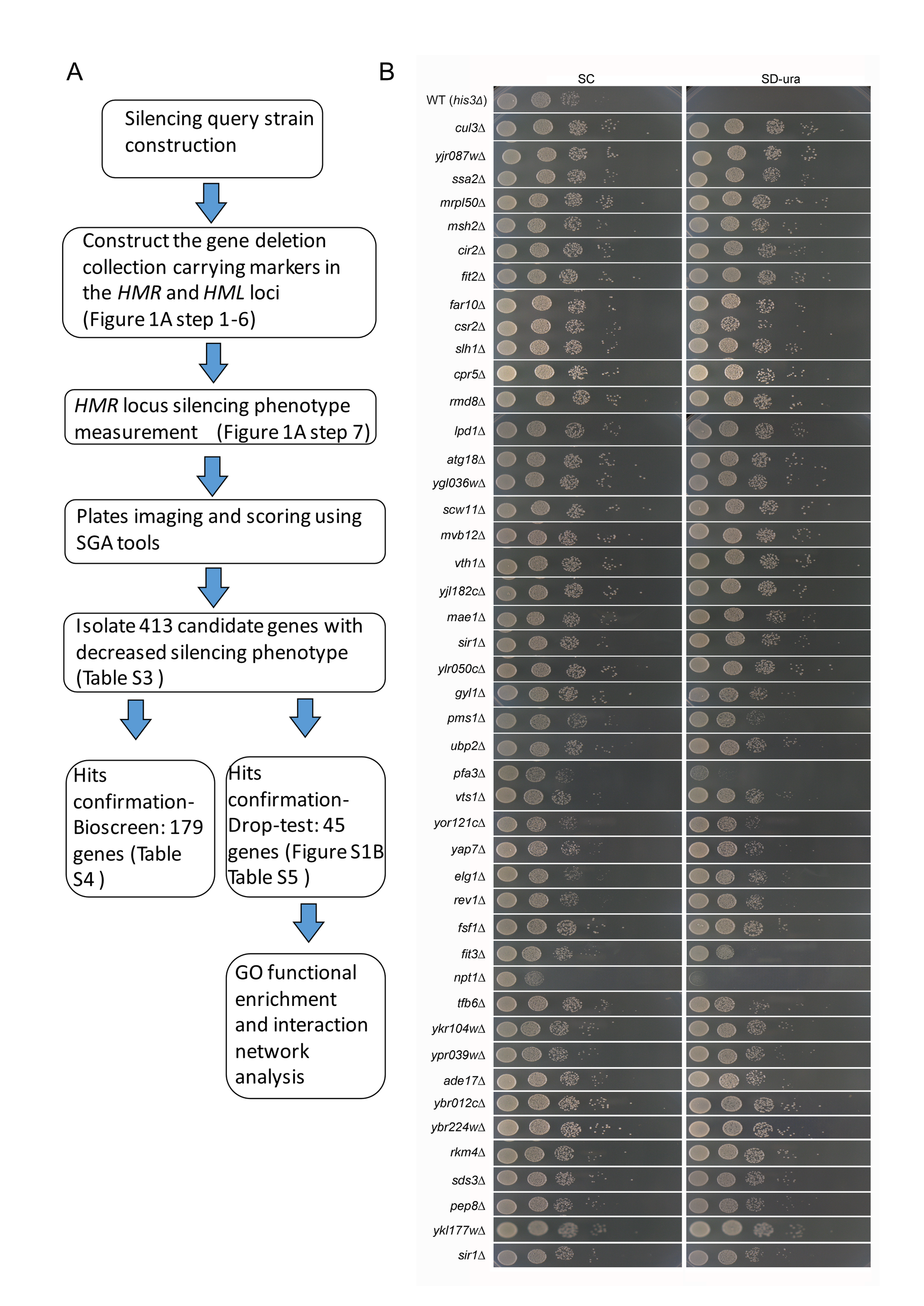

Supplement: S1 Fig — A flow diagram of the overall silencing screening procedure (A) and Spot tests confirmed the decreased mating-type silencing phenotypes of the deletion mutants identified from the silencing screen (B). Cells were 10-fold serially diluted and then spotted onto SC (left) and SD-Ura (right) agar plates; the sir1Δ mutant served as a positive control. (TIF) [file pgen.1008798.s001.tif]

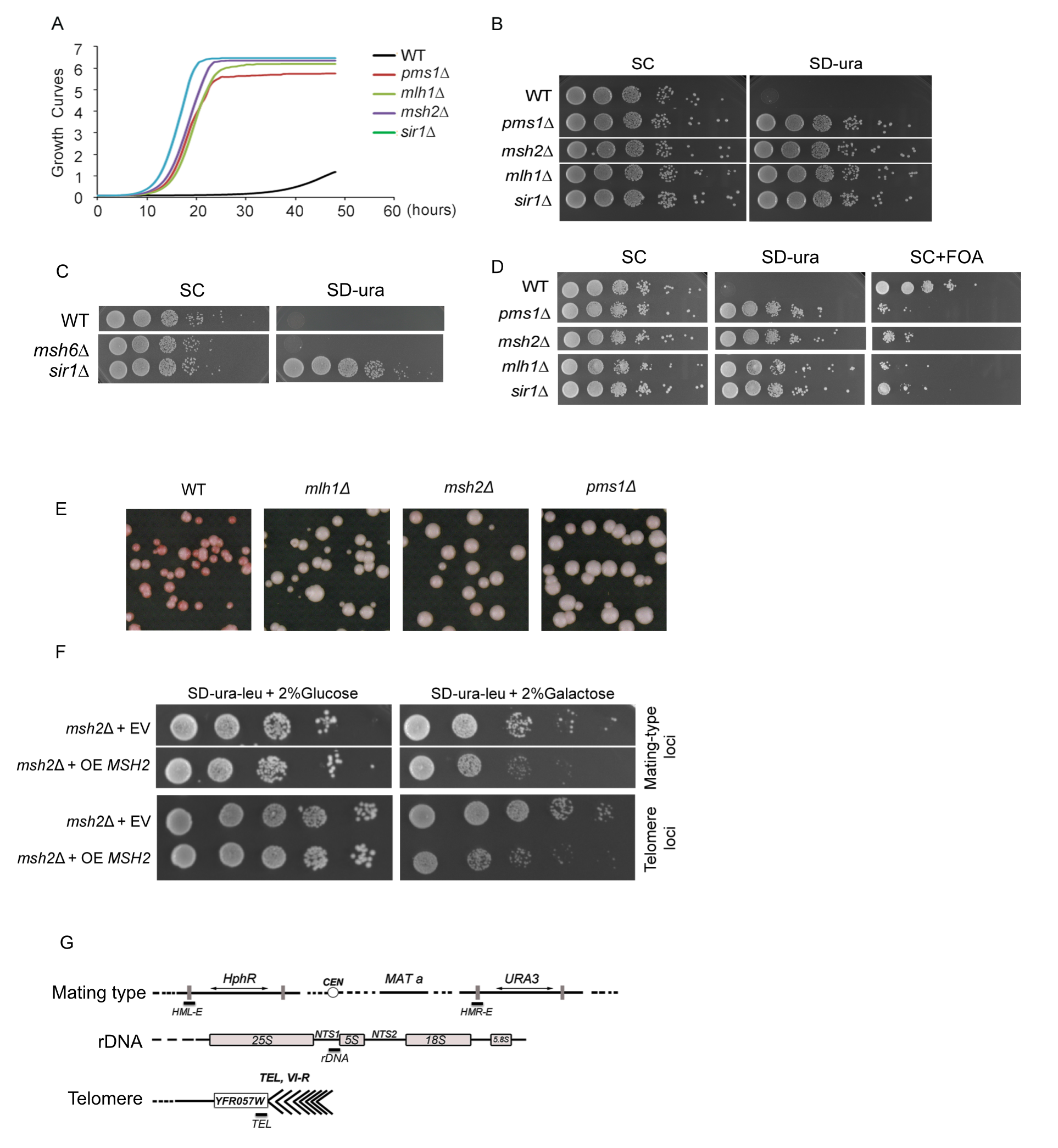

Supplement: S2 Fig — (A) MMR mutants have increased growth rates in SD-Ura liquid medium. The growth rates were measured using the Bioscreen mini-liquid culture approach. (B) Confirmation of pms1Δ, mlh1Δ, and msh2Δ decreased mating-type silencing phenotype by PCR knockout using the natMX4 marker. The sir1Δ mutant obtained from the silencing screen was used as a positive control. (C) Deletion of MMR component gene MSH6 didn’t affect mating type silencing. sir1Δ mutant was used as a positive control. (D) Cells of indicated strains were 10-fold serially diluted and then spotted onto SC (left), SD-Ura (middle) and SC+FOA(right) agar plates, the sir1Δ mutant served as a positive control. (E) Loss of silencing at telomere ADE2 reporter visualized by red color formation. WT (UCC3505) and the corresponding MMR mutants were grown on YPD medium. MMR deletion strains (pms1Δ, mlh1Δ, and msh2Δ) displayed white with sectors colonies as compared to WT, indicating a loss of gene silencing at the telomere ADE2 reporter. Cells were five-fold serially diluted and grown at 30℃ followed by storage in 4℃ until clear red pigment formation could be seen (15 days). (F) Decreased telomere silencing in the MSH2 mutants were rescued by overexpressed MSH2. The plasmids (pRS425 and pRS425-Gal10-MSH2) were transformed to msh2Δ mutants in both WT (his3Δ) and UCC3505 backgrounds. Cells were five-fold serially diluted and then spotted onto SD-ura-leu + 2% glucose (left) or SD-ura-leu + 2% galactose (right) agar plates. Overexpressed MSH2 (OE MSH2) in the msh2Δ mutants partially restore telomere and mating type silencing, as compared to overexpressed empty vector (EV). (G) Schematic diagram showing the position of primers corresponding to: HMR (HMR-E) and HML (HML-E) loci on chromosome III, the rDNA (NTS1/2) on chromosome VII, and the TEL (YFR057W) on the right arm of chromosome VI. These primers were used in chromatin immunoprecipitation (ChIP) experiments and gene expression (YFR057W). (TIF) [file pgen.1008798.s002.tif]

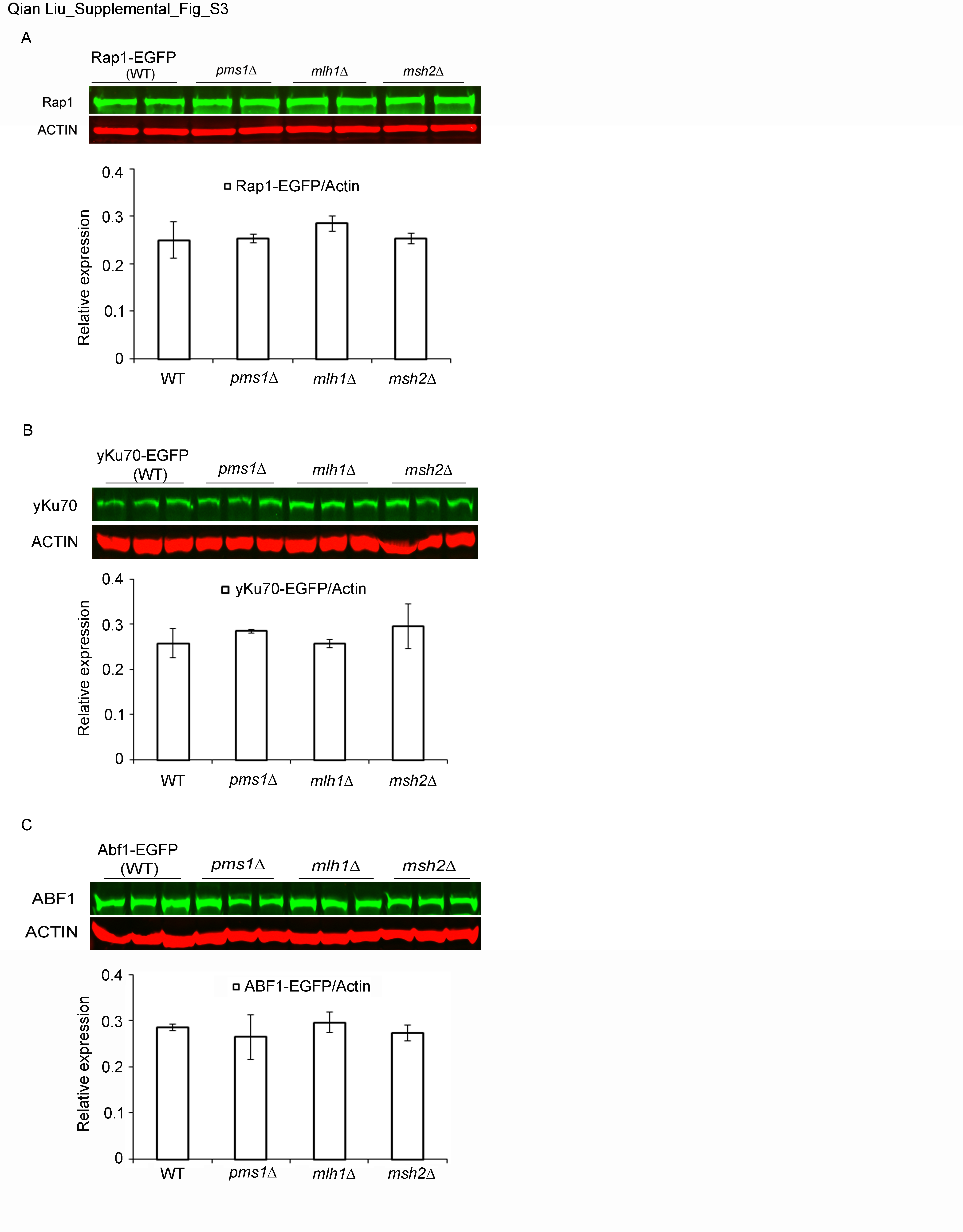

Supplement: S3 Fig — Western blot analysis using GFP antibody revealed no significant changes in the protein levels of Rap1 (A), yKu70 (B), or Abf1 (C) in the MMR deletion mutants compared to the WT. (TIF) [file pgen.1008798.s003.tif]

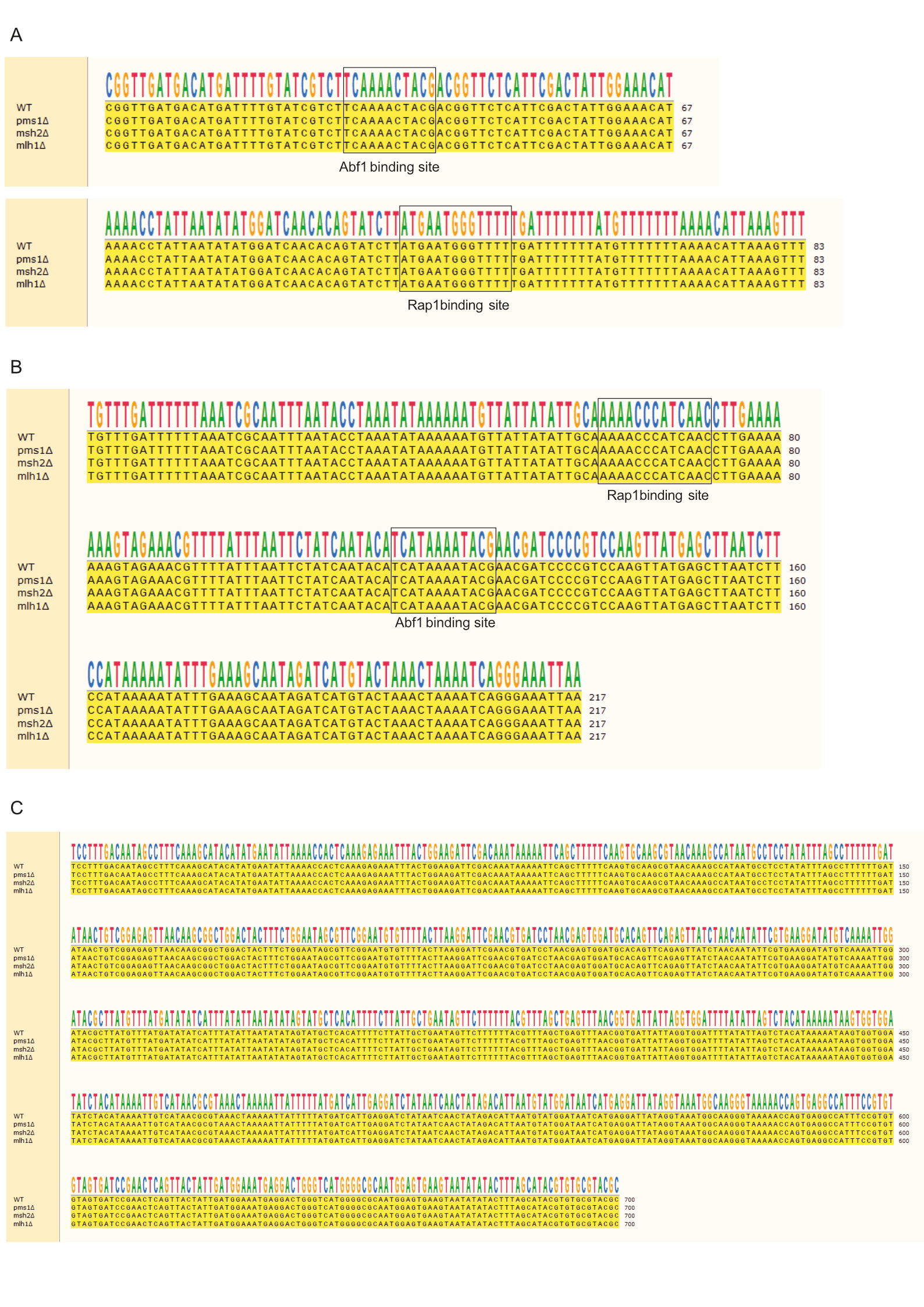

Supplement: S4 Fig — Multiple sequence alignments of the MMR mutants (pms1Δ, msh2Δ and mlh1Δ) compared to WT strain using the Align Multiple DNA Sequences tool in SnapGene. A. Sequencing results of the binding sites of Abf1 (top) and Rap1 (bottom) in the HML locus. B. Sequencing results of the HMR locus with the binding sites of Rap1 and Abf1 highlighted. C. Sequencing results of the Tel6R region. Yellow color indicates matching bases. (TIF) [file pgen.1008798.s004.tif]
